# Consensus Modeling Strategies for Predicting Transthyretin Binding Affinity from Tox24 Challenge Data

**DOI:** 10.1021/acs.chemrestox.5c00018

**Published:** 2025-05-15

**Authors:** Thalita Cirino, Luis Pinto, Mateusz Iwan, Alexis Dougha, Bono Lučić, Antonija Kraljević, Zaven Navoyan, Ani Tevosyan, Hrach Yeghiazaryan, Lusine Khondkaryan, Narek Abelyan, Vahe Atoyan, Nelly Babayan, Yuma Iwashita, Kyosuke Kimura, Tomoya Komasaka, Koki Shishido, Taichi Nakamura, Mizuho Asada, Sankalp Jain, Alexey V. Zakharov, Haobo Wang, Wenjia Liu, Vladimir Chupakhin, Yoshihiro Uesawa

**Affiliations:** † Molecular Biotechnology and Health Sciences Department, 9314 University of Turin, Turin 10126, Italy; ‡ Independent Researcher, Montreal H2Y 3Z2, Canada; § 9361 Mario Negri Institute for Pharmacological Research IRCCS, Milan 20156, Italy; ∥ BFA, 555089 Université Paris Cité, CNRS UMR 8251, Inserm U1133, Paris 75013, France; ⊥ 54583 Ruđer Bosković Institute, Zagreb 10000, Croatia; # Faculty of Mechanical Engineering, Computing and Electrical Engineering, University of Mostar, Mostar 88000, Bosnia and Herzegovina; ∇ Toxometris.ai., Glendale, California 91204 United States; ° Institute of Molecular Biology, NAS RA, Yerevan 0014, Armenia; ◆ Biocentric.ai., Yerevan 0075, Armenia; ¶ Department of Medical Molecular Informatics, 34779 Meiji Pharmaceutical University, Tokyo 204-8588, Japan; †† 390834 National Center for Advancing Translational Sciences (NCATS−NIH), Rockville, Maryland 20850, United States; ‡‡ Key Laboratory of Industrial Ecology and Environmental Engineering (Ministry of Education), Dalian Key Laboratory on Chemicals Risk Control and Pollution Prevention Technology, School of Environmental Science and Technology, 12399 Dalian University of Technology, Dalian 116024, China; §§ 264970 Cheminformatics Solutions, Simulations Plus, Lancaster, California 93534, United States

## Abstract

Transthyretin (TTR) is a key transporter of the thyroid hormone thyroxine, and chemicals that bind to TTR, displacing the hormone, can disrupt the endocrine system, even at low concentrations. This study evaluates computational modeling strategies developed during the Tox24 Challenge, using a data set of 1512 compounds tested for TTR binding affinity. Individual models from nine top-performing teams were analyzed for performance and uncertainty using regression metrics and applicability domains (AD). Consensus models were developed by averaging predictions across these models, with and without consideration of their ADs. While applying AD constraints in individual models generally improved external prediction accuracy (at the expense of reduced chemical space coverage), it had limited additional benefit for consensus models. Results showed that consensus models outperformed individual models, achieving a root-mean-square error (RMSE) of 19.8% on the test set, compared to an average RMSE of 20.9% for the nine individual models. Outliers consistently identified in several of these models indicate potential experimental artifacts and/or activity cliffs, requiring further investigation. Substructure importance analysis revealed that models prioritized different chemical features, and consensus averaging harmonized these divergent perspectives. These findings highlight the value of consensus modeling in improving predictive performance and addressing model limitations. Future work should focus on expanding chemical space coverage and refining experimental data sets to support public health protection.

## Graphical abstract

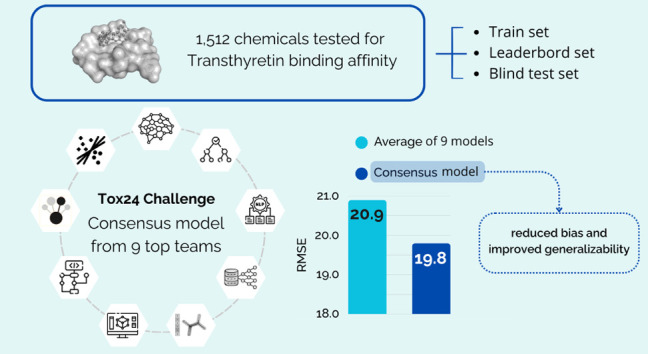

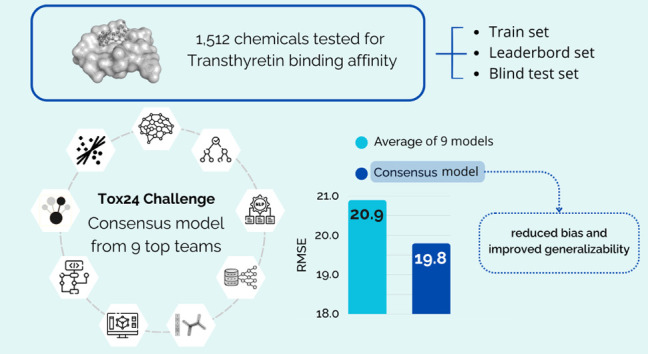

## Introduction

Endocrine-disrupting chemicals (EDCs) represent a significant concern for public health and industrial chemical safety due to their prevalence in the environment. They can affect the endocrine system even at low concentrations by mimicking natural hormones, blocking hormone receptors, and interfering with thyroid hormones’ transport, among others.^1^ Previous studies^2,3^ have demonstrated that EDCs can bind to transthyretin (TTR), a protein responsible for transporting thyroxine (T4),^4^ which reduces the availability of this hormone.

High-throughput (HTP) assays are widely employed to identify or quantify activity of potential EDCs and provide an alternative to *in vivo* models.^5,6^ Although these assays enable rapid assessment of thousands of chemicals at various concentrations, screening large chemical libraries remains costly and time-consuming. This is particularly challenging for thyroid system disruption assessment due to its complexity and multiple potential targets.^7^

To overcome these limitations, researchers have turned to *in-silico* approaches, such as molecular docking or QSAR models.^8^ The latter can predict binding affinity based on molecular structure and physicochemical properties, but require a sufficiently large and diverse data set during training. However, individual models are often inherently biased. The No Free Lunch theorem states that no single algorithm is optimal for every problem or application. This is especially true in cheminformatics, where the vast diversity of chemical space makes it hard for one model to generalize well. A previous study^9^ demonstrated that consensus modeling, which averages predictions from multiple models, enhances prediction quality. This is achieved by mitigating individual models’ bias while expanding the applicability domain (AD).^10^

The standard deviation of predictions from multiple models (Consensus-STD) as a Distance-to-Model (DM) metric is frequently used to assess model uncertainty, and was shown to be the most effective approach for toxicity predictions by Tetko et al.^9^ High Consensus-STD values often correlate with low quality predictions and typically occur for compounds outside the chemical space of the training data set. However, a combination of low Consensus-STDs and high prediction errors may hint at the presence of outliers – compounds that deviate significantly from the expected trends despite being within the chemical space of the training data set. These may originate from errors in the chemical representation, difficulties in experimental measurements^11^ (*e.g.*, interference with assay, low solubility or stability), or happen due to the presence of activity cliffs.^12^

The Tox24 Challenge,^13^ inspired by the successful Tox21 Challenge,^14^ aimed to advance computational toxicology by evaluating novel *in-silico* approaches for predicting chemical binding to TTR, based on a curated data set from a recent HTP assay.^15^ Building on these efforts, we developed a consensus model by combining individual models from nine top-performing Tox24 Challenge teams. We also identified potential outliers and substructures that impact prediction quality. This work advances chemical space analysis and modeling and may support regulatory decision-making in chemical safety assessment and contribute to more effective public health protection regulations.

## Data

### Data Source

The data set used in the study was generated using a fluorescence-based *in vitro* screening assay that measured the binding affinity to TTR^15^ by assessing the displacement of 8-anilino-1-naphthalenesulfonic acid (ANSA) from human TTR. ANSA was used as a fluorescent probe, and its displacement from TTR by test compounds caused a measurable decrease in fluorescence intensity. The fluorescence changes were measured and compared to a reference curve created using T4. The results were expressed as a percentage of ANSA displacement, with higher T4 concentrations representing stronger binding to TTR. Compounds that showed autofluorescence or produced inconsistent biological results were excluded, resulting in a final data set of 1512 compounds.^13,15^

### Challenge Format

The Online Chemical Modeling Environment (OCHEM)^16^ website (https://ochem.eu) was the host of the Tox24 Challenge. The challenge organizers provided compound names, CAS numbers, and chemical structures as Simplified Molecular Input Line Entry Specification (SMILES) notation. The latter was primarily sourced from the U.S. EPA’s CompTox Chemicals Dashboard (https://comptox.epa.gov/dashboard), with PubChem based on CASRN or DSSTOX identifiers serving as a secondary source.

After identifying compounds that overlapped with previous studies,^17–19^ the challenge organizers split the data set so that all previously known compounds were assigned to the training set, ensuring no data leakage during model validation:

1. A training set containing 1012 compounds.
2. A leaderboard set comprising 200 compounds.
3. A blind test set of 300 compounds.

During the first stage of the challenge, the training set was used to develop models, while the leaderboard served as a validation set with unknown binding affinity data. Fifteen days before the end of the challenge, the leaderboard set was released and could be incorporated into the training set, depending on whether the teams chose to use it. For clarity, any references to the *test set* in this document refer to the blind test set, which was kept confidential throughout the whole challenge.

### Data Analysis

Figure 1 shows the distribution of TTR binding affinities across the training (before including the leaderboard), the leaderboard, and the blind test set, expressed as activity percentage (%) corresponding to the displacement of the ANSA probe. The values range from −41.6 to 110.9%, with most compounds in all three data sets located in the 0–30% TTR binding affinity region. Each subset contains a similar fraction of compounds across toxicity value ranges, ensuring consistency in the activity distributions.

**Figure 1:**
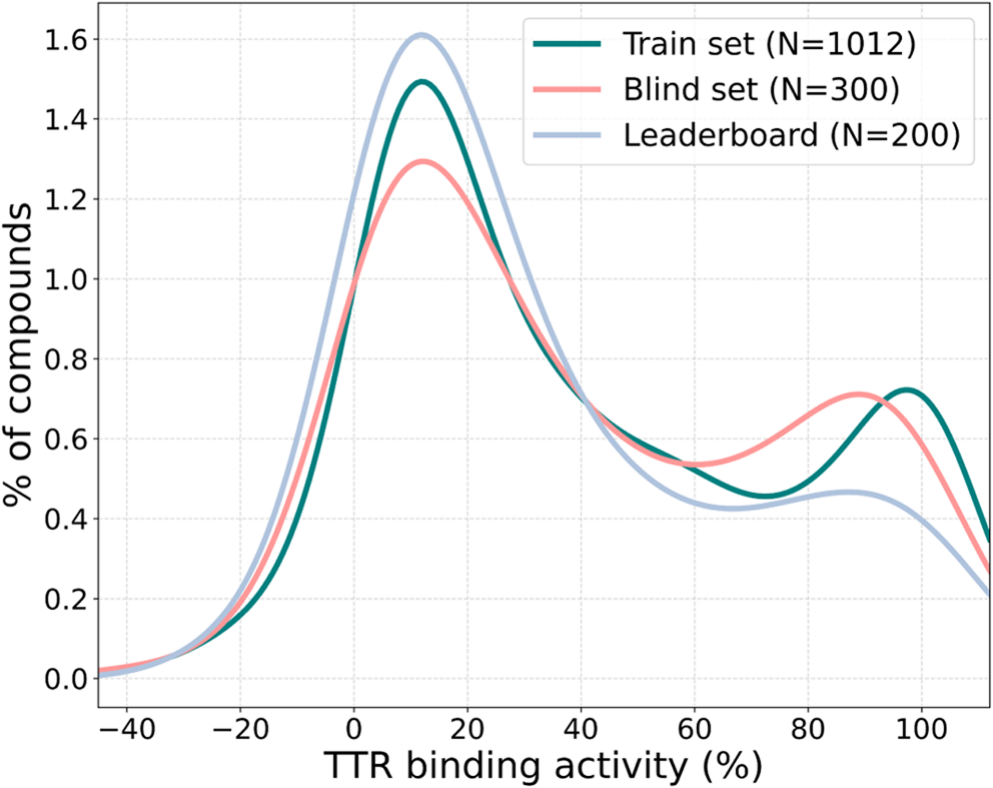
Distribution plot of the experimental TTR binding activity for the training set (in green), leaderboard set (in blue), and blind test set (in red).

The empirical cumulative distribution function (eCDF) of the maximum and mean Tanimoto similarity between test and training compounds was calculated using Morgan fingerprint (radius of 2, 1024-bit-long).^20^ Figure 2A shows that most compounds in both the leaderboard and blind sets exhibit moderate similarity to at least one training compound (0.4–0.8). However, a significant portion of both sets displays low similarity (≤0.4), indicating structural diversity. While the blind set contains a higher proportion of highly similar compounds (0.8–1.0), the mean similarity for both sets is low (Figure 2B), suggesting that, despite some close analogs, most test compounds are structurally distinct from the majority of the training set.

**Figure 2:**
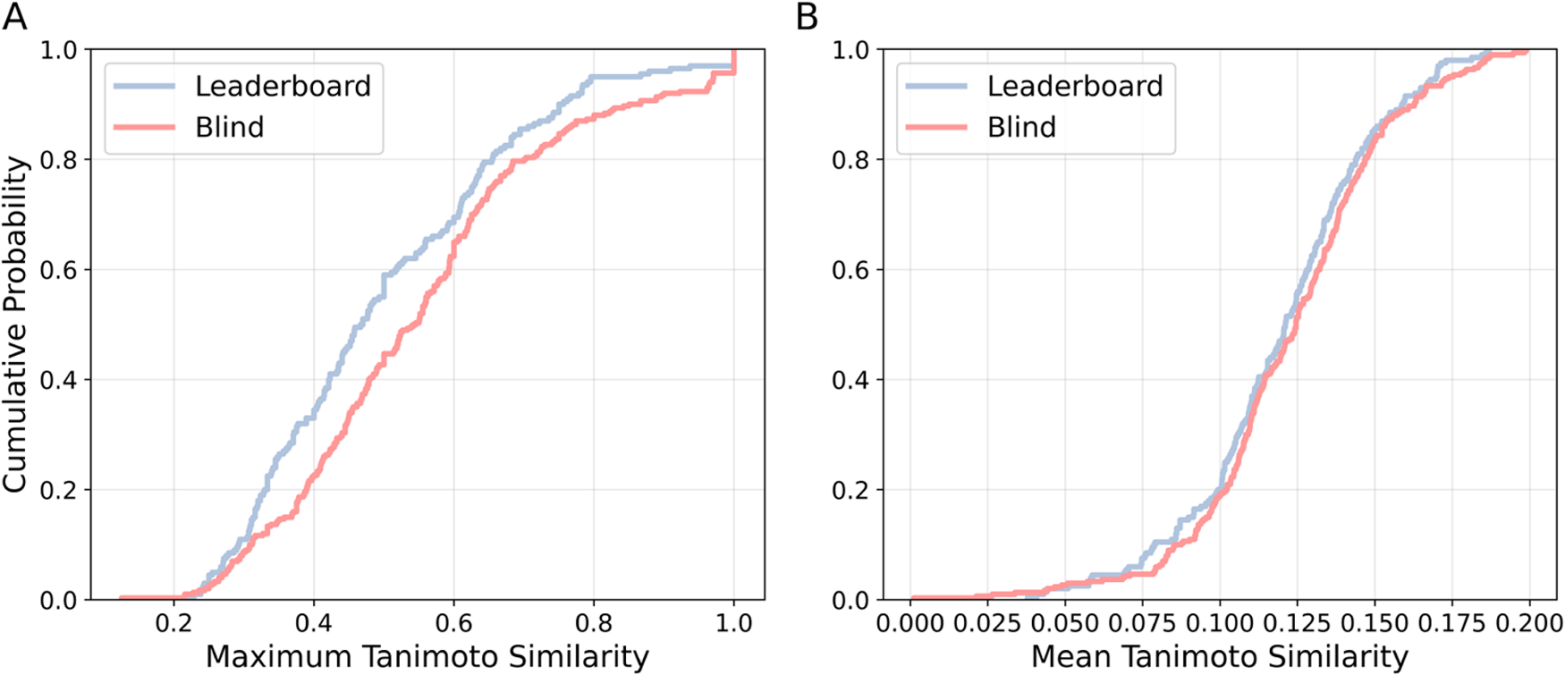
Empirical cumulative distribution functions (ECDF) of Tanimoto similarities for both leaderboard (blue) and blind test (red) sets. Plot A displays the maximum Tanimoto similarities, while plot B shows the mean Tanimoto similarities.

An analysis was conducted using the SetCompare tool in OCHEM^16^ to identify functional groups overrepresented in the active subset. As reported in the experimental study,^15^ 888 chemicals were considered active for exhibiting more than 20% activity compared to the high T4 concentration control in single-concentration screening. Overrepresentation was assessed using two criteria: (1) a statistical significance threshold of *P* < .01 and (2) the enrichment factor (EF, calculated as the ratio of a functional group’s percentage in the active set to its percentage in the inactive set) higher than 2.5. Overrepresented functional groups, their EFs and *P*-values are shown in Table 1.

**Table 1:** Functional Groups Overrepresented in Active Compounds, Showing: The Number of Compounds Containing Each Group in Both Active and Inactive Subsets, Their EF, and *P*-Value of Enrichment

functional group	active	inactive	EF	*P*
phenols	157	9	12.2	<.001
thiophosphoric acid derivatives	29	3	6.7	<.01
nitro compounds	56	7	5.6	<.001
diarylethers	40	5	5.6	<.001
benzyl halides	42	7	4.2	<.001
gem-trihalides	72	13	3.9	<.001
primary aromatic amines	53	11	3.4	<.001
aryl halides	185	50	2.6	<.001
arenes	609	168	2.5	<.001

The analysis revealed that functional groups such as phenols, aryl halides, and diarylethers were significantly enriched in the active subset. While these functional groups are also present in the structure of T4, the analysis also identified other functional groups not present in T4 but that appear to play a role in activity. For example, groups such as nitro compounds and thiophosphoric acid derivatives showed significant enrichment in the active subset, suggesting that binding is not strictly limited to structural similarity with T4.

This observed complexity in structure–activity relationships, where both T4-like and non-T4-like functional groups contribute to binding, demonstrates the need for more sophisticated tools. The diverse chemical space present in the data set provided makes it particularly well-suited for machine learning algorithms, which can systematically identify and quantify the complex relationships between chemical structures and biological activity.

## Methodology

In this study, we analyzed computational models for TTR binding affinity developed by nine teams among the top 11 from the Tox24 Challenge—with an RMSE not statistically different from that of the first place solution.^21^ We aimed to evaluate and optimize consensus modeling strategies by exploring different methods of averaging predictions from multiple models. The following subsections detail the procedures for individual model development, evaluation metrics used to assess performance, and consensus modeling strategies across these scenarios.

### Regression Metrics

To evaluate model performance, we employed three standard statistical metrics: the squared cross-validation correlation coefficient for the training set (*Q* ^2^), the coefficient of determination for the test set (*R* ^2^), and the root-mean-square error (RMSE)—eq 1. Both *Q* ^2^ and *R* ^2^, eq 2, quantify the proportion of variance explained by the model, with *Q* ^2^ calculated during cross-validation on the training set and *R* ^2^ calculated on the test set. The RMSE measures the average deviation between predicted and observed values. All metrics were calculated using the percentage of TTR binding activity (*Y*) as the target variable.

1 $$\mathrm{RMSE}=\sqrt{\frac{1}{N}\sum_{Y}{\left(Y_{\exp}-Y_{\mathrm{pred}}\right)}^{2}}$$

2 $$Q^{2},R^{2}=1-\frac{\sum_{Y}{\left(Y_{\exp}-Y_{\mathrm{pred}}\right)}^{2}}{\sum_{Y}{\left(Y_{\exp}-{\mathrm{Y̅}}_{\exp}\right)}^{2}}$$

### Individual Model Development and Analysis

Table 2 summarizes the data preprocessing steps, modeling approaches, and applicability domain (AD) definitions used by each participating team, ordered by their final ranking based on the RMSE of their last submission; the model descriptions correspond to those that generated the final set of predictions submitted in the Tox21 Challenge.^21^ Detailed descriptions are available in the Supporting Information.

#### Modeling Approach

The nine teams employed diverse data preprocessing and modeling strategies. While some teams relied solely on single-method approaches (employing either only descriptor-based methods or only representation learning methods), five teams (#2, #3, #6, #7, and #9) employed consensus models that integrated both descriptor-based and representation learning methods. Teams #2 and #6 developed their models using the OCHEM platform, with both implementations publicly available at https://ochem.eu/article/160931.

Among descriptor-based approaches, the most commonly used were ensemble decision tree-based algorithms, including Random Forest (RF),^22^ Categorical Boosting (CatBoost),^23^ eXtreme Gradient Boosting (XGBoost),^24^ and Light Gradient-Boosting Machine (LGBM).^25^ Other descriptor-based methods, used less frequently, included Kernel Partial Least Squares (KPLS),^26^ Deep Learning Consensus Architecture (DLCA),^27^ and Support Vector Machines (SVM).^28^ Representation learning approaches were divided into two main categories: SMILES-based and graph-based algorithms. SMILES-based methods included Convolutional Neural Networks (CNNs), Transformer CNN^29^ and Convolutional Neural Fingerprint (CNF2),^30^ MolFormer,^31^ and SMILE Transformer Encoder Decoder (SMI-TED).^32^ Graph-based models comprised Graph Neural Networks (GNNs), such as Attentive Fingerprint (AttFP)^33^ and ChemProp,^34^ implemented via Keras Graph Convolutional Neural Networks (KGCNN),^35^ as well as Graph Attention Networks (GAT).^36^

**Table 2:** Summary of Modeling Approaches Used by the Nine Groups, from Data Preprocessing to AD Definition

ranking (RMSE)	data preprocessing	modeling approach	AD definition
#2 tcirino (20.7%)	OCHEM standardization, charge neutralization and salt remover. RDKit^38^ data augmentation with tautomers. Outlier filtering.^39^	consensus of ten models: Transformer CNN and CNF2, KGCNN (ChemProp and AttFP), KPLS with Mordred(2D)^40^ descriptors, and five CatBoost, each with a different set of 2D descriptors.^37,38,40–42^ All implemented in OCHEM (https://ochem.eu/model/1160)	consensus model predictions standard deviation
#3 znavoyan (20.7%)	salt-stripped SMILES. Duplicates averaged if target values differed <50% or had ≥ 3 values; otherwise excluded.	consensus of three models in an ensemble: two RF based −one with RDKit and the other with OEstate descriptors,^43,44^ both combined with bioassay descriptors; and one GNN using multilevel features and docking data.	ensemble mean absolute deviation.
#4 Microsomes (20.8%)	descriptors were calculated on two types of SMILES: one with desalination preprocessing applied and the other without it.	meta-learning approach: Base models (XGBoost, RF, CatBoost, LGBM) were stacked by combining their predictions with descriptors (Mordred and RDKit) to train a final XGBoost model.	minimal Euclidean distance to training compounds.
#6 AntonijaBoss (21.2%)	train set without leaderboard. OCHEM standardization, charge neutralization and salt remover.	two models consensus: one RF with AlogPS^45^ + CDK23 ^46^ descriptors and another CNF2. Model available in https://ochem.eu/model/1207	consensus model standard deviation
#7 SankalpJain (21.3%)	Knime implementation of Atkinson standardizer.^47^	DLCA combining fingerprints (Morgan, Avalon, and AtomPair), RDKit physicochemical descriptors and Convolutional Neural Network based on SMILES.	Tanimoto similarity using Morgan fingerprints.^20^
#8 alx.dga (21.4%)	Salt-stripped SMILES.	consensus models (SVM and RF-based) chosen based on Tanimoto similarity to training data, using RDKit and Knowledge-Guided Pretraining of Graph Transformer latent descriptors.^48^	consensus model predictions standard deviation
#9 luispintoc (21.4%)	OCHEM standardization and charge neutralization. RDKit canonicalization. Averaged target values for identical canonical SMILES	Hill climbing ensemble (MolFormer, SMI-TED, and UniMol^49^) Auxiliary target: tree-based models (LGBM and CatBoost) predictions using molecular descriptors.^37,43^	consensus model predictions standard deviation
#10 Wang (21.4%)	RDKit canonicalization followed by SaltRemover to strip counterions and small solvent molecules.	transfer learning based on GAT architecture.	similarity density and inconsistency of activity.^50^
#11 vchupakhin (21.4%)	RDKit standardization.	ensemble of CatBoost Voting Regressors.^51^	standard deviation of the ensemble predictions.

Although the model descriptions in Table 2 correspond to those that generated the last submissions in the Tox21 Challenge, three teams made additional refinements for the final consensus: team #2 selected a subset of four models – Transformer CNN, CNF2, KGCNN-ChemProp, and CatBoost trained with Mold2 ^37^ descriptors—out of ten for averaging, reducing the blind test RMSE from 20.7 to 20.3%. Similarly, team #9 achieved a reduction in their RMSE (21.4 → 20.4%) by removing the MolFormer^31^ model trained with CatBoost predictions; this configuration corresponded to their penultimate submission, which outperformed their last one. Meanwhile, team #6 improved their performance (RMSE: 21.2 → 20.5%) by incorporating the leaderboard set into their training. This selection was motivated by the fact that these models were available to partners during the competition, although they were not submitted as final entries for various reasons.

#### Model Validation

Most teams employed a k-fold cross-validation (CV) protocol. Team #4 implemented a nested-CV, and team #10 used a Monte Carlo CV with 20 random splits. In nested-CV, an inner loop is used for model optimization while an outer loop provides unbiased performance estimation, offering an additional layer of validation robustness. The Monte Carlo CV approach, while ensuring independence between training and test sets in each split, differs from traditional k-fold CV as observations might appear multiple times or never in the test sets due to the random nature of the splits.

### Consensus Modeling

We implemented two consensus approaches based on the treatment of ADs: nonweighted averaging of (I) all individual model predictions regardless of their ADs, and (II) only predictions from models that had the compound within their AD. For approach II, a compound was considered within the AD as long as at least one contributing model included it within its AD. Additionally, we tested different stringency levels by progressively increasing the minimum number of models required to consider a compound within the AD of the consensus model, from at least two to all models.

Because our consensus included predictions that were themselves from consensus models or ensembles, the total number of underlying individual models exceeds 20, with the potential to reach significantly higher counts when accounting for all base submodels (e.g., team #11’s 50-model ensemble).

#### Potential Errors Detection

Five teams (#2, #3, #6, #8, and #9) developed consensus models. The provided Consensus-STD values were used as a DM metric^9^ to assess model uncertainty. An automated outlier detection algorithm was implemented, following the methodology described by Tetko et al.^39^ Due to differences in CV protocols, each consensus model was analyzed individually.

The algorithm was based on a bin-based averaging (BBA)^52^ approach that measures how prediction accuracy changes with increasing DM. Using the CV predictions and Consensus-STDs as the basis for binning, the BBA process divides DM values into nonoverlapping intervals (referred to as bins). Within each bin, prediction accuracy is calculated, and a bin is finalized when two criteria are met: (1) it contains at least 50 compounds, and (2) the accuracy of the bin is lower than that of the preceding bin. The latter criterion is because the accuracy is expected to decrease (or at least not improve) as DM increases, reflecting the relationship between uncertainty and prediction accuracy. The BBA plot is often paired with a residuals plot (commonly referred to as a Williams plot) to visualize the spread of prediction errors and further support outlier detection. A data point is flagged as an outlier when its prediction error exceeds the threshold defined for the bin it belongs to.

#### Substructure Importance Analysis

A postmodeling substructure importance analysis was conducted to examine the correlation between the presence of specific substructures and RMSE values. Klekota and Roth fingerprints^53^ were calculated for the blind test set; only substructures present in at least 32 entries were kept for further analysis. We defined the null hypothesis (*H*_0_) as presence of substructure *X* does not affect prediction quality. For each substructure, the subset of compounds containing it was selected, and the RMSE_sub_ was calculated. To simulate *H*_0_, the predictions squared errors were adjusted so that the RMSE_sub_ matched that of the test set. 100,000 bootstrap samples were simulated to obtain the RMSE distribution under the null hypothesis (RMSE_bs_). A *P*-value was calculated as a fraction of RMSE_bs_ values lower than (improvement) or greater than (degradation) RMSE_sub_. We used *P* <.05 as a significance threshold. To account for the multiple hypotheses being tested we applied the Benjamini-Hochberg (BH) procedure to control the false discovery rate.^54^

## Results and Discussion

### Statistical Evaluation of Individual Models

Table 3 presents the statistical metrics for the nine models (one per group) on both the training set (*Q* ^2^ and RMSE) and blind test set predictions (*R* ^2^ and RMSE), without (I) and with (II) considering ADs. RMSE values ranged from 20.3 to 21.4% on the test set without considering AD. While some models (#2, #6 and #9) described in Table 3 outperform the Tox24 challenge winning model (https://ochem.eu/article/162082), such comparisons require context: these models were preferred over the one corresponding to the last submission after the blind test set disclosure. Similarly, the winning team later reported that, by including a Chemprop-based^34,35^ model in their consensus, the RMSE dropped from 20.7 to 20.3%.^55^ However, this enhancement may reflect adjustments informed by the test set rather than true generalization.

**Table 3:** Statistical Parameters of Models Combined in the Consensus, Including Train Set CV and Blind Test Set Validation^a^

				blind test set (*N* = 300)				
	train set CV			no AD (I)		with AD (II)		
model	*Q* ^2^	RMSE (%)	data set size	*R* ^2^	RMSE (%)	*R* ^2^	RMSE (%)	% within AD
#2^b^	0.61	22.6	1212	0.68	20.3	0.68	19.8	88.3
#3	0.64	21.6	1195	0.67	20.7	0.71	19.1	75.3
#4	0.57	23.5	1212	0.66	20.8	0.68	20.3	92.0
#6^b^	0.63	22.7	1212	0.67	20.5	0.67	20.0	91.7
#7	0.51	25.4	943	0.64	21.3	0.70	20.0	51.0
#8	0.55	24.2	1199	0.65	21.4	0.67	20.3	83.3
#9^b^	0.66	20.8	1145	0.67	20.4	0.79	13.8	75.3
#10	0.48	26.5	1212	0.64	21.4	0.68	20.4	91.3
#11	^c^	^c^	^c^	0.64	21.4	0.66	20.8	90.0
consensus				0.69	19.8	0.69	19.8	100.0

a For the latter, two consensus approaches are reported: without AD (Consensus I) and with AD (Consensus II).

b Models metrics are different from last submission reported in Eytcheson and Tetko.^21^

c Metric not included due to inconsistency in validation methodology.

For all individual models, the implementation of ADs enhanced prediction accuracy, leading to lower RMSE values. The percentage of compounds within each model’s AD varied significantly (51–92%), indicating diversity of AD definitions. For instance, model #7, with the most restrictive AD (51.0% coverage), experienced a modest improvement in RMSE (1.3 %). In contrast, model #9, with a more inclusive AD (75.3% coverage), achieved a substantial reduction in RMSE (4.5 %).

Most teams relied on representation learning methods, which are expected to excel due to their ability to learn intrinsic patterns and relationships directly from raw molecular data, without relying on explicit descriptor engineering. This capability mitigates information loss, which is particularly valuable when working with small data sets. Among descriptor-based approaches, the ensemble decision tree-based algorithms dominated, as they can effectively model non-linear relationships and feature interactions, even with limited data.

### Performance of Consensus Models

Blind test predictions were aggregated into two consensus approaches (I and II), as shown in Table 3 and explained in the Methodology Section. Consensus I achieved an RMSE of 19.8 compared to an average RMSE of 20.9 across individual models without AD. Interestingly, the consensus approaches showed no difference in RMSE whether AD was applied or not, and so the consensus methods may already compensate for AD variability by effectively aggregating predictions across multiple models.

Figure 3 presents the RMSE of predictions as a function of TTR binding activity for individual models and the consensus model (Consensus I). The Consensus I line is smoother and often lies at or below the other lines, indicating better predictive performance overall. Across all models, the RMSE is lowest for moderate binding activities (10–40%) and increases at the extremes of the experimental range. This trend suggests that the models are more reliable in the central activity range, likely due to a higher density of training data in this region. Additionally, at the lower extreme (up to 10%), the increased RMSE may reflect the challenges of accurately quantifying weak binders or nonbinders, where noise introduced by low solubility and aggregation (among other assay artifacts) may lead to inconsistently low activity readings.

**Figure 3:**
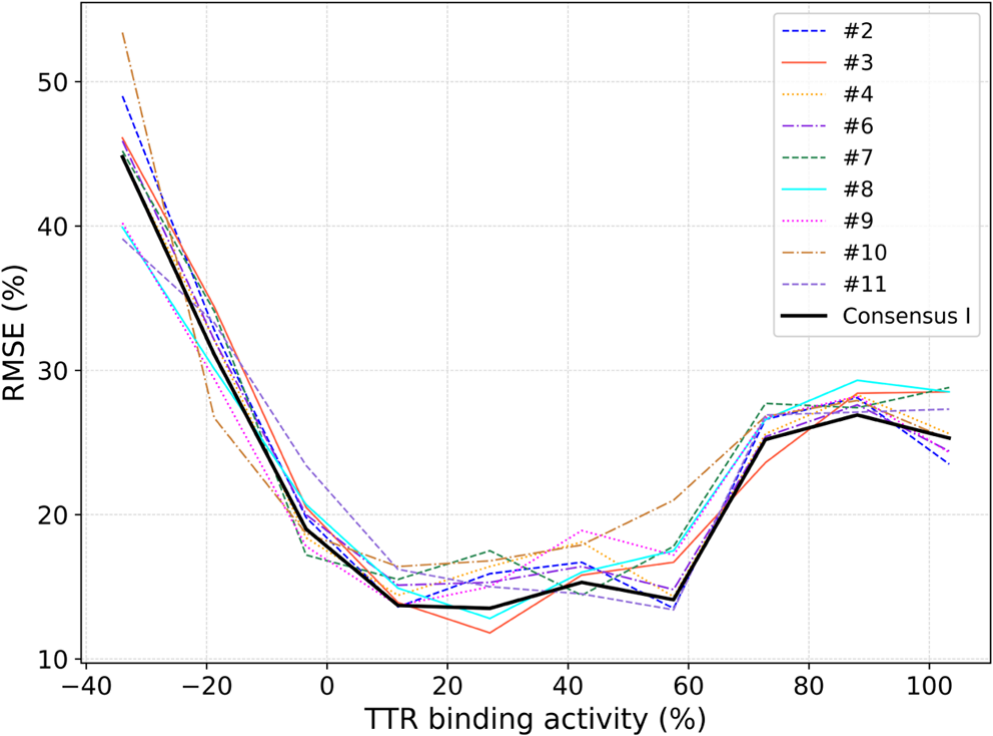
RMSE of predictions as a function of TTR binding activity (%) for each team model and the consensus model (Consensus I).

Table 4 presents the trade-off between prediction reliability and coverage. The most permissive approach, requiring predictions to be within the AD of at least one model, provided 100% coverage. In contrast, the most conservative approach, requiring predictions to be within the AD of all nine models, achieved a significantly lower RMSE of 13.7% but reduced coverage to only 28.3%. This trade-off underscores the challenge of balancing predictive accuracy with broad applicability.

**Table 4:** Statistical Parameters for Consensus II Models Performance over the Validation Set from Most Permissive (with at Least 2 Predictions) to Most Conservative (with All 9 Predictions) Criteria

	consensus II		
minimal predictions	*R* ^2^	RMSE (%)	% coverage
2	0.69	19.8	100.0
3	0.70	19.7	99.3
4	0.70	19.7	99.3
5	0.70	19.6	96.7
6	0.74	17.9	89.0
7	0.77	16.8	77.7
8	0.79	15.7	54.0
9	0.84	13.7	28.3

### Model Selection for Consensus Modeling

CV metrics typically guide model selection for consensus building. As shown in Table 3, the best CV RMSE performance (95% CI: [19.6, 22.1]) was obtained by Team #9’s model. A comparison of confidence intervals can suggest which models have a not significantly different performance and can be considered statistically equivalent. Specifically, models #3 ([20.6, 22.6]), #2 ([21.4, 23.9]), and #6 ([21.6, 24.0]) exhibited overlapping confidence intervals with model #9. Conversely, models with a lower bound exceeding 22.1% were excluded as their RMSE values were significantly higher. The consensus built from these four models achieved the same performance as the one built out of the nine models. This suggests that predictive accuracy is nearing the limits imposed by the inherent experimental uncertainty in the data set. The minimal impact on RMSE when refining the consensus model by excluding predictions with large deviations (based on Grubbs’ statistics at a significance level of 0.1) further supports this reasoning.

Since the CV metrics were obtained over a data set five times larger than the leaderboard set, they are more stable and representative of model performance. However, if the CV protocol is not carefully made, its results can be overoptimistic.^56^ Similarly, teams optimizing for leaderboard performance risked overfitting over this data set, reducing generalizability. Teams optimizing for leaderboard performance risked overfitting, compromising generalizability. This is supported by two key comparisons: (1) A strong correlation (Pearson *r* = 0.85) between CV RMSE and blind-test RMSE after the leaderboard release (Figure 4A and Table 3); and (2) a weak correlation (*r* = 0.37) between leaderboard-set RMSE and blind-test RMSE prior to the leaderboard release (Figure 4B), with both metrics derived from models trained without access to the leaderboard data, as reported by Eytcheson and Tetko.^21^ In contrast, our consensus model shows consistency: an RMSE of 19.8% was obtained for both the leaderboard and the blind test sets. This highlights the approach’s stability and effectiveness in the correction of variance and bias of each model while improving overall performance.

**Figure 4:**
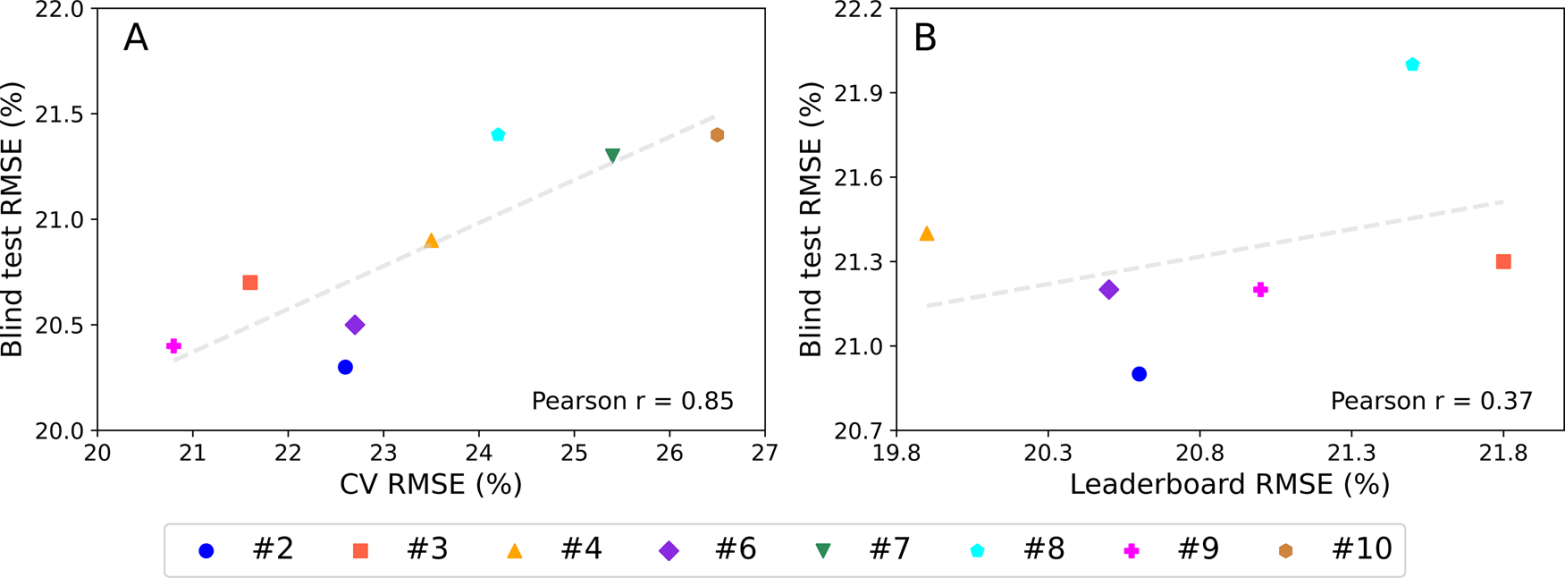
Scatter plots showing (A) Blind test and CV RMSE (%) of the models combined in the consensus (Table 3). (B) Blind test and Leaderboard RMSE (%) as submitted to the challenge before the release of the leaderboard set. Each point represents a team, color-coded consistently across both plots. Dashed trend lines indicate Pearson correlation coefficients.

The relationship between RMSE of CV and blind test predictions reflects how different protocols can significantly affect model performance assessment during development. For example, teams #2 and #6 used OCHEM’s protocol, which employs InChI-based splitting and separate hyperparameter selection, preventing overoptimistic metrics—comparable to team #4’s nested CV, which was explicitly designed to mitigate overfitting. These strategies preserve test fold independence, which is critical since parameter tuning or feature selection on the full data set prior to CV can yield misleading metrics.^56^ To address the limitation that OCHEM’s InChI-based fold splitting might fail to distinguish tautomeric forms,^57^ team #2 excluded augmented data during model validation to prevent overfitting in their CV metrics. However, this validation protocol does not fully represent the final model’s performance, as it omits the augmented data incorporated in the trained models that generated the actual predictions.

As further discussed in the Substructure Importance Analysis Section, heterogeneity in model architectures and data preprocessing strategies contributes to more generalizable consensus predictions by averaging out inherent biases introduced during model selection. Thus, beyond standard CV and validation on intermediate benchmark sets, the selection of models should prioritize methodological diversity, including the preprocessing step, to enhance model capability to generalize.

When experimental noise is substantial, even sophisticated models or consensus approaches cannot reach the experimental accuracy. This underscores the importance of acknowledging the underlying data quality when interpreting RMSE values and assessing potential for further model refinement. In this case, the consensus model serves primarily to stabilize predictions rather than achieve dramatic accuracy gains.

### Identifying Potential Errors in the Data through Consensus Model

Figure 5 presents the Williams plot for five teams (#2, #3, #6, #8 and #9), highlighting a common pattern: the majority of predictions cluster in regions of low Consensus-STD with corresponding low prediction errors. Notable exceptions are the identified outliers, which show unexpectedly high prediction errors despite their low Consensus-STD values. This pattern suggests that while the models are generally reliable, there are specific compounds where high confidence predictions (indicated by low Consensus-STD) do not align with actual performance, warranting further investigation of these cases.

**Figure 5:**
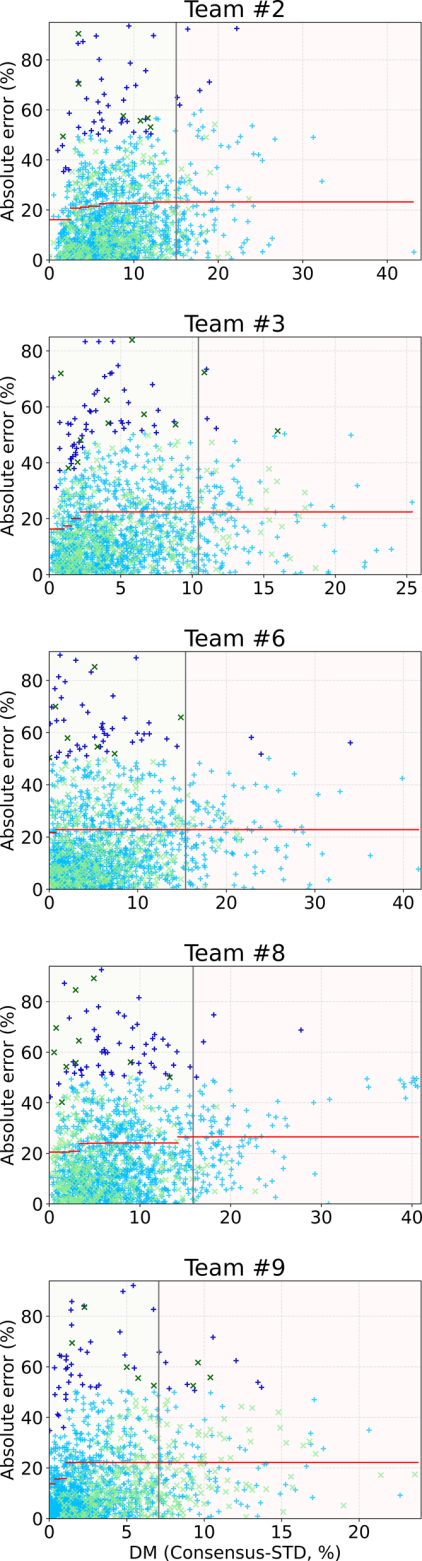
Williams plots for the five consensus models (from teams #2, #3, #6, #8, and #9), showing the relationship between absolute prediction errors and Consensus-STD values. Each plot includes both training (light blue ‘+’) and test (light green ‘*x*’) data points, with outliers marked in darker colors. The red lines represent bin-averaged RMSE values, establishing expected error thresholds for different Consensus-STD ranges. The vertical line indicates the AD threshold (at 90% of the training set).

For each model training set, an average of 50 outliers were identified, corresponding to less than 5% of the respective training set. The CV RMSEs of these outliers ranged from 52.8 to 67.9%, and excluding these outliers resulted in a noticeable improvement in models’ performance, as shown in Table 5. Despite the differences in training protocols and architectures, 45 outliers in the whole set were identified for at least three models and are more likely to represent true outliers rather than random noise. Detailed information about identified outliers is available in the Supporting Information (see SI2_OutliersAnalysis.xlsx file).

**Table 5:** Cross-Validation Metrics for the Training Set Excluding Outliers and for Outliers Alone, Including the Size of Each Set

	train set CV (outliers excluded)			outliers CV	
model	*Q* ^2^	RMSE (%)	Size	RMSE (%)	Size
#2	0.84	14.0	1167	67.9	45
#3	0.73	18.3	1139	56.7	55
#6	0.70	19.4	1166	63.8	46
#8	0.65	20.6	1143	61.9	56
#9	0.88	12.2	1117	52.8	45

While filtering out these outlying data points generally increases the prediction performance also over the blind set, it is important to note that this conclusion cannot be fully verified without retraining the models.

### Substructure Importance Analysis

Initially, each model identified different substructures as important, indicating that models learn different features they consider important for predictions. This variation may also be influenced by differences in the preprocessing steps of each team. By averaging across these diverse perspectives, the consensus model seems to reflect the middle ground across other models, which may contribute to its improved performance. However, after applying the BH procedure to control for false positives, only four statistically significant substructures remained. Notably, these substructures were shared among most models. These four substructures—a tetrahedral carbon atom with a methyl group as one of the substituents, a sulfur atom with two bonds, an isopropanol scaffold, and an ethyl formate scaffold - showed a statistically significant correlation (*P* < .05) with improved prediction quality. Further details on the statistical significance analysis of substructures are provided in the (see SI).

## Conclusions

This study demonstrates the effectiveness of consensus modeling strategies in predicting TTR binding affinity using data from the Tox24 Challenge. By combining predictions from multiple individual models, we improved the performance, with the best consensus approach achieving an RMSE of 19.8% in both leaderboard and blind test sets. The implementation of AD enhanced individual model performance, but the benefits were not observed in consensus models, suggesting that this approach inherently compensates for model uncertainties.

The substructure importance analysis revealed that each model identified different substructures as important, reflecting differences in preprocessing steps and model-specific learning mechanisms. However, by combining these diverse perspectives, the consensus model provides a broader range of relevant features, which likely contributes to its improved performance. Future directions could include exploring Explainable AI (XAI) methods to better investigate what each model has learned. XAI is a set of techniques that can be used to explain the reasoning behind model predictions or make them understandable for humans. Although this field needs to be matured,^58^ the fast development of representation learning approaches and the emergence of novel architectures that can provide explanation of models,^59^ promise more accurate and interpretable predictions to further support more reliable toxicity assessments and risk evaluations.

The systematic identification of outliers identified compounds that require further experimental investigation, potentially indicating either experimental artifacts or cases of activity cliffs. Therefore, future work should focus on expanding the chemical space coverage and investigating the identified outliers to enhance both model performance and data quality.

Ultimately, our results support the value of consensus modeling in toxicological predictions while acknowledging the limitations imposed by experimental uncertainty. This work provides a framework for future computational toxicology studies and could support regulatory decision-making in chemical safety assessment.

## Supplementary Material







## Notes

Detailed overview of the approach taken by each team. The teams are listed and described as they appear in the tables presented earlier (PDF)
Full data set containing indicators (True/False) for outlier detection in five teams (#2, #3, #6, #8, and #9) and a frequency count (0–5) indicating in how many models each compound was identified as an outlier (XLSX)
Detailed statistical information for the four significant molecular fragments, including the number of significant substructures identified by each model before and after the BH correction (), the specific molecular fragments that significantly affected prediction quality with their corresponding *P*-values (), and 95% confidence intervals for each statistically significant fragment showing the RMSE for relevant test data set subsets () (PDF)
