# Supplementary material for: Consensus Modeling Strategies for Predicting Transthyretin Binding Affinity from Tox24 Challenge Data
Source: Chem Res Toxicol. 2025 May 15;38(6):1061–71. doi: 10.1021/acs.chemrestox.5c00018 (PMC12175157; doi:10.1021/acs.chemrestox.5c00018)
Supplement: Supplementary file 1 [file tx5c00018_si_001.pdf]

Supplementary Information 1

Consensus Modeling Strategies for Predicting Transthyretin Binding Affinity from Tox24 Challenge Data

Thalita Cirino,[*,†] Luis Pinto,[‡] Mateusz Iwan,[¶] Alexis Dougha,[§] Bono Lučić,[‖] Antonija Kraljević,[⊥] Zaven Navoyan,[#] Ani Tevosyan,[#] Hrach Yeghiazaryan,[#] Lusine Khondkaryan,[#,@] Narek Abelyan,[△] Vahe Atoyan,[#] Nelly Babayan,[#,@] Yuma Iwashita,[▽] Kyosuke Kimura,[▽] Tomoya Komasaka,[▽] Koki Shishido,[▽] Taichi Nakamura,[▽] Mizuho Asada,[▽] Sankalp Jain,[††] Alexey V. Zakharov,[††] Haobo Wang,[‡‡] Wenjia Liu,[‡‡] Vladimir Chupakhin,[¶¶] and Yoshihiro Uesawa[▽]

†*Molecular Biotechnology and Health Sciences Department, University of Turin, 10126 Turin, Italy*

‡*Independent Researcher. Montreal, Canada*

¶*Mario Negri Institute for Pharmacological Research IRCCS, 20156 Milan, Italy*

§*BFA, Université Paris Cité, CNRS UMR 8251, Inserm U1133, 75013 Paris, France*

‖*Ruđer Bosković Institute, 10000 Zagreb, Croatia*

⊥*Faculty of Mechanical Engineering, Computing and Electrical Engineering, University of Mostar, 88000 Mostar, Bosnia and Herzegovina*

#*Toxometris.ai. Glendale, CA 91204 United States*

@*Institute of Molecular Biology, NAS RA. Yerevan 0014, Armenia*

△*Biocentric.ai. Yerevan 0075, Armenia*

▽*Department of Medical Molecular Informatics, Meiji Pharmaceutical University. Tokyo 204-8588, Japan*

††*National Center for Advancing Translational Sciences (NCATS–NIH). Rockville, MD 20850 USA*

‡‡*School of Environmental Science and Technology, Dalian University of Technology. Dalian 116024, China*

¶¶*Cheminformatics Solutions, Simulations Plus. Lancaster, CA 93534 USA*

E-mail: thalita.cirino@unito.it

This SI provides a detailed overview of the approach taken by each of the eight participating teams. The teams are listed and described as they appear in the tables presented in the main text.

Table of Contents

Team #2 . . . . **S-3**

Team #3 . . . . **S-6**

Team #4 . . . . **S-8**

Team #6 . . . . **S-11**

Team #7 . . . . **S-13**

Team #8 . . . . **S-16**

Team #9 . . . . **S-19**

Team #10 . . . . **S-21**

Team #11 . . . . **S-25**

References **S-26**

Team #2

Thalita Cirino

Molecular Biotechnology and Health Sciences Department, University of Torino, Italy

In the Tox24 Challenge,[S1] I achieved 2nd place among 79 participants, with my final submission attaining an RMSE of 20.7% on the blind test set. My modeling approach centered on two main strategies: (1) data augmentation via tautomer generation and (2) consensus modeling. During the challenge, I combined predictions from ten models through unweighted averaging. Post-challenge analysis revealed that selecting the four most structurally diverse models for the consensus further reduced the blind-test RMSE to 20.3%. Notably, similar to the winning team's use of mixture descriptors,[S2] my implementation of data augmentation (specifically tautomer enumeration) during preprocessing aimed to provide a more comprehensive representation of the chemical space.

Data Preprocessing

Compounds were standardized through the OCHEM[S3] preprocessing built-in tools, which includes (I) standardization by converting structures into canonical SMILES and correcting functional groups (e.g., nitro and azido groups) to a single chemical representation; (II) charge neutralization by attaching additional hydrogen atoms; and (III) counterions and small solvent molecule removal, returning only the bigger fragment in mixtures.

Data augmentation. To broaden the representation of the chemical space, tautomeric forms of each compound were generated using the TautomerEnumerator class in RDKit 2020.03.1.[S4] Tautomerism, a form of structural isomerism where compounds can exist in different forms through the migration of a hydrogen atom and reorganization of double bonds, can significantly influence molecular properties and interactions.[S5] As experimental activity data for individual tautomeric forms was not available, the assigned TTR binding activities were inherited from the parent structures. While this approach cannot account for poten-

tial differences in binding affinity between tautomers, more rigorous analysis would require estimating dominant solution-phase forms (via quantum or molecular mechanics energy calculations with Boltzmann weighting) and assessing protein-bound conformations (through molecular dynamics simulations). Such methods could reveal whether minor tautomeric species contribute disproportionately to binding activity, but these investigations remain beyond the scope of the current study.

Outliers filtering. The consensus standard deviation (Consensus-STD) was used to exclude outlying train data. We used the same procedure described by Tetko et al.,[S6] where errors which were unlikely to be produced by mixture of gaussian distribution were identified and excluded.

Model Development

The final predictions were obtained through unweighted averaging of four selected models, differing from the challenge submission's ten-model consensus. This post-challenge optimization focused not only on 5-fold cross-validation (5CV) performance (as in the original submission), but also on maximizing algorithmic diversity. The final consensus incorporated:

(I) CatBoost[S7] - *Mold2*[S8], a gradient-boosting algorithm combined to a set of 2D molecular descriptors;

(II) Keras Graph Convolutional Neural Network (KGCNN) ChemProp,[S9,S10] a graph-based algorithm that has each node receiving messages from its neighbors to then update its representation based on the aggregated message;

(III) Transformer Convolutional Neural Networks (TransformerCNN),[S11] a Natural Language Processing method that was pre-trained over 1.7M molecules from the ChEMBL database[S12] to learn the task of canonisation of chemical structures. The learned latent representation is used as input to one-dimensional Convolutional Neural Network (CNN) and its output is correlated with the target properties of molecules using fully connected neural networks;

(IV) Transformer Convolutional Neural Network Fingerprint (CNF2)[S13] extends the previous method and uses a combination of several CNNs with different receptive fields to provide a richer representation (fingerprint) to be correlated with target properties of molecules.

Applicability Domain

The predictions from the four selected models were combined through simple (non-weighted) averaging to produce the final predictions, while the applicability domain (AD) study was carried out based on the Consensus-STD.[S14] Its value covering 85% of data in the training set was used as the threshold to determine the AD threshold of the model.

Team #3

Zaven Navoyan[1], **Ani Tevosyan**[1], **Hrach Yeghiazaryan**[1], **Lusine Khondkaryan**[1,3], **Narek Abelyan**[2], **Vahe Atoyan**[1], **Nelly Babayan**[1,3]

[1] *Toxometris.ai, Glendale, CA 91204, USA*

[2] *Biocentric.ai, Yerevan 0075, Armenia*

[3] *Institute of Molecular Biology, NAS RA, Yerevan 0014, Armenia*

In order to achieve superior results, several techniques described below are used, each of which improves RMSE by a different factor. Techniques that were tried but did not lead to a significant improvement in results are not included.

- Train random forest (RF) models[S15] on bioassay data from ToxCast and eMolTox, then use these models to make predictions on data from the Tox24 Challenge. Subsequently, use these predictions as descriptors in the main models. The bioassay data preprocessing and model training are fully automated, e.g. automatically detecting and filtering nan containing data points, low variance data etc. In cases where the bioassay data is unbalanced, different balancing techniques are automatically applied based on the degree of imbalance. For highly imbalanced cases (imbalance ratio $<$ 10%), undersampling is used. For moderate imbalance, the SMOTE balancing technique[S16] is applied. When the number of data points in both classes is nearly equal, no balancing is used. The trained models are then validated to ensure they have an AUC greater than a specified threshold.
- The best-performing descriptors for RF were selected using a Genetic Algorithm.[S17]
- Different models are used in an ensemble, and the final prediction is calculated by averaging the predictions of the individual models within the ensemble. This allows for capturing different aspects of molecular representations. We used the following models:

- RF based on RDKit[S4] descriptors + Bioassay descriptors,
- RF based on MACCS fingerprints[S18]
- Graph Neural Network (GNN).

- Use of Specialized GNN: We utilize a GNN with a residual block architecture, similar to ResNet.[S19] Up to 30 different GNN kernels were tested, and ultimately, PNA (Principal Neighborhood Aggregation) was chosen as the best solution for this problem. The GNN used RDKit and bioassay features at node, edge, and graph levels, enhanced with molecular docking data.
- Multitask setup: Molecular docking was performed for compounds from the dataset and the calculated binding score was used along with other bioassay descriptors.

Team #4

Yoshihiro Uesawa, Yuma Iwashita, Kyosuke Kimura, Tomoya Komasaka, Koki Shishido, Taichi Nakamura, Mizuho Asada

Department of Medical Molecular Informatics, Meiji Pharmaceutical University. Tokyo 204-8588, Japan

To participate in this competition, our laboratory established a prediction model development team named microsomes. This presentation introduces our approach to constructing a predictive model for TTR binding affinity scores.

Methodology

Descriptor Generation. For machine learning descriptors, we utilized the descriptor dataset provided by the organizers, along with descriptors generated using Mordred[S20] and RDKit.[S4] Calculations with Mordred and RDKit were performed on two types of SMILES: one with desalination preprocessing applied and the other without it. Additionally, we incorporated prediction probabilities for 59 molecular initiating events as features, which were obtained using the Toxicity Predictor[S21] developed in our laboratory. Descriptors and features with zero variance among compounds or missing values were removed. To eliminate multicollinearity, when pairs of descriptors had an R^2 value of 1, one descriptor from each pair was removed.

Modeling Scheme. In this study, machine learning models were constructed using decision tree-based algorithms. Initially, training was conducted using 4-fold CV for four base models: XGBoost,[S22] LightGBM,[S23] CatBoost,[S7] and RF.[S15] To create a stacking model integrating these base models, a dataset was prepared by combining the original descriptors with the prediction outputs from the base models. This dataset was then trained using XGBoost with nested CV (Figure S1-A). For nested CV, the outer loop was divided into 4 folds, and the inner loop into 3 folds, to evaluate and optimize the hyperparameters.

Evaluation Metrics. The competition utilized the root mean square error (RMSE) as the metric to evaluate the prediction accuracy of the models for the compounds. The winning team was determined based on the RMSE for the Blind dataset. The model development period in the competition was divided into two phases, separated by the public release of the experimental values for the Leaderboard dataset on August 15, 2024. Before the release, models from participating teams were ranked based on the RMSE for the Leaderboard dataset, allowing for a relative evaluation of prediction accuracy. Based on the RMSE for the Leaderboard dataset, we established the descriptors and modeling scheme to be used (Figure S1-A). After the release of the experimental values, the Leaderboard dataset was combined with the Train dataset, and models were constructed following the established methodology to predict the TTR binding affinity scores for the compounds in the Blind dataset.

Results and Discussion

The total number of descriptors calculated during the dataset preparation process for machine learning was 3,976. After cleaning, including the elimination of multicollinearity, 1,655 descriptors were selected for model construction. Prior to the public release of the experimental values for the Leaderboard dataset, our team achieved the top rank among 51 teams (a total of 943 models) in the competition. Figure S1-B illustrates the scatter plots of the TTR binding affinity scores versus the predicted values from our model for the Leaderboard and Blind datasets. At this stage, the RMSE for the Leaderboard dataset was 19.9. For the Blind dataset, the RMSE of our predictions was 20.8, placing us 4th among 79 teams. Notably, the RMSE of the winning team's model was 20.5, and there was no statistically significant difference between our model and theirs. As a result, the models developed by the top 11 teams, including ours, were recognized as the Top Scoring Models. Since the competition required predictions for all compounds in the Blind dataset, no applicability domain was established. We believe that the appropriate utilization of applicability domains, such

as by calculating inter-compound distances, could further improve the predictive accuracy of our model.

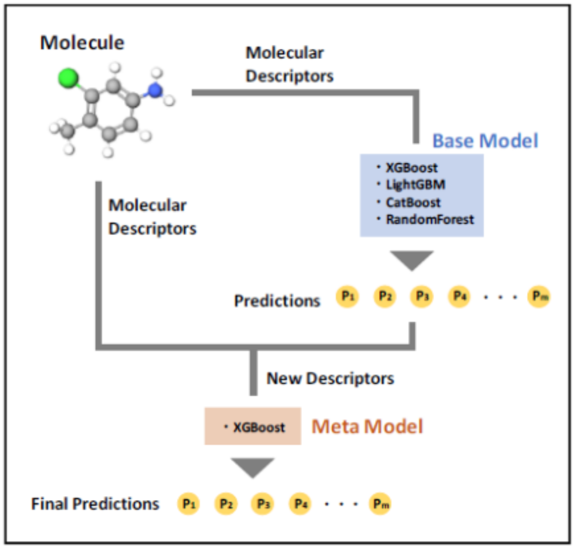

Figure S1-A: Modeling scheme.

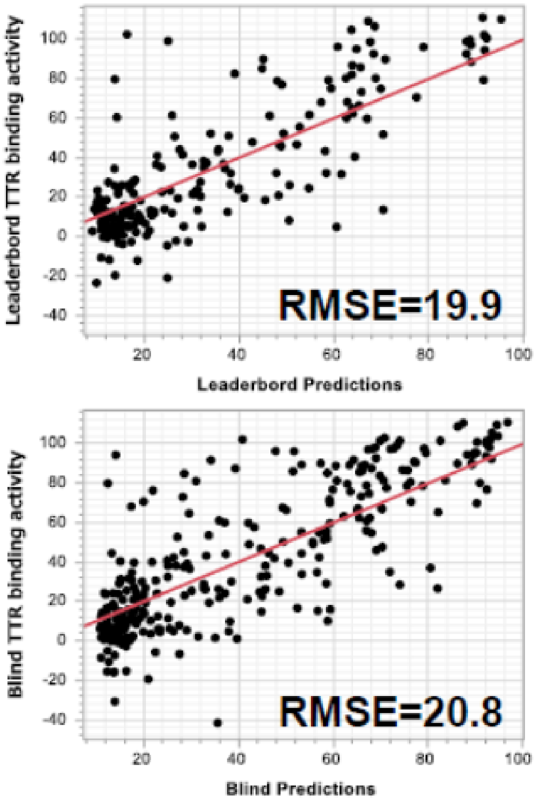

Figure S1-B: Plots of observed versus predicted values for the leaderboard and test set.

Team #6

Antonija Kraljević[1], Bono Lučić[2]

[1] *University of Mostar, Faculty of Mechanical Engineering, Computing and Electrical Engineering, Mostar, Bosnia and Herzegovina*

[2] *Ruđer Bošković Institute, NMR Centre, Zagreb, Croatia*

Our intention was to develop simple consensus models for predicting TTR binding activity using various Machine Learning (ML) algorithms available on the OCHEM platform.[S3] These included algorithms that utilize structural information encoded in molecular descriptors – e.g., RF[S15] – and representation learning algorithms that extract structural information directly from the SMILES representation.

Data Sets and Model Selection:

Initially, individual models and a consensus model were developed using a training set of 1012 molecules. To assess the performance of different ML methods in OCHEM, a 5CV protocol was applied. Based on its results, the best-performing models were selected for further evaluation using a leaderboard set of 200 compounds. The final single and consensus models were chosen based on the minimum RMSE.

Final individual and consensus models:

For the final blind set prediction, the consensus model was rebuilt using an expanded training set of 1212 compounds, incorporating the original 1012 training molecules along with the 200 leaderboard compounds. The final prediction was based on a consensus of only two individual models: (1) RF Regression using OEstate,[S24,S25] and CDK23[S26] descriptors, and (2) Transformer Convolutional Neural Network Fingerprint (CNF2),[S13] the same described above by Team #2.

Results

Incorporating the leaderboard set improved our model's performance from 21.2% RMSE (last submission at ochem.eu/model/1207) to 20.5% RMSE on the blind-test set (N = 300). This improved model, whose performance is comparable to the winning submission to the challenge,[S27] was selected for inclusion in our final consensus in this study.

Applicability Domain

As done by Team #2, the Consensus-STD value which covered 90% of data in the training set was used as AD threshold of the model.

Team #7

Jain Sankalp, Alexey V. Zakharov

National Center for Advancing Translational Sciences (NCATS) – NIH. Rockville, MD 20850 USA

Data Curation

Chemical standardization is a critical first step in obtaining a high-quality dataset for predictive modeling. This process ensures that all molecules are consistently represented, reducing data noise and improving the reliability of the model's predictions. In this study, the chemical structures of molecules in both the training and test sets were standardized and curated following established data curation practices using KNIME.[S28] Briefly, this involved stripping salts and solvents from all compounds, followed by the removal of counterions, large organic molecules (with more than 100 heavy atoms), mixtures, and inorganic compounds. During this process, some compounds were found to have identical chemical representations after standardization. To handle these cases systematically, we applied a threshold-based approach for duplicate entries. If duplicate compounds exhibited discordant potency values (i.e., activity differences $> 10\%$), both entries were excluded to avoid introducing artificial noise into the dataset. If the potency values were within this threshold, an average was calculated, and a single entry was retained to prevent over-representation.

Molecular Descriptor

In this study, we utilized a combination of molecular descriptors and representations to enhance model performance. Three types of molecular fingerprints were employed: Morgan, Avalon, and AtomPair. Each of these fingerprints encodes unique aspects of molecular structure. Morgan fingerprints capture local substructures, Avalon fingerprints efficiently encode functional groups and stereochemistry, and AtomPair fingerprints focus on spatial relationships between atom pairs.

We also integrated RDKit physicochemical descriptors, which provide a comprehensive numerical representation of key molecular properties such as molecular weight, logP, polar surface area, and hydrogen bond donors/acceptors. These descriptors are crucial for modeling ADMET properties.

In addition to descriptor-based models, we implemented and evaluated a descriptor-free approach using SMILES notation. This encoding allows us to utilize deep learning techniques, particularly convolutional neural networks (CNNs), which can learn patterns from the linear string representation of molecules. By combining these methods, we created a hybrid framework that captures both explicit molecular features and deep structural patterns, leading to improved prediction accuracy across various tasks.

Machine Learning Methods

In this study, we employed a "Deep Learning Consensus Architecture" (DLCA),[S29] which integrates outputs from multiple deep neural networks (DNNs) into a unified framework. Each DNN was constructed using distinct molecular descriptors, and by averaging their outputs, the DLCA approach aims to enhance prediction accuracy while minimizing error propagation between models.

Our implementation combines both descriptor-based and descriptor-free models within the DLCA framework. Descriptor-based models were generated using three types of molecular fingerprints—Morgan, Avalon, and AtomPair—along with RDKit physicochemical descriptors. For the descriptor-free model, SMILES notation was employed to encode molecular structures, with a convolutional neural network (CNN) architecture utilizing 1D convolutional and GlobalMaxPooling layers. By leveraging this consensus approach, the DLCA model benefited from the complementary strengths of each descriptor and fingerprint type, resulting in a more robust and accurate predictive model. While SMILES-based models have been shown to provide strong performance in other contexts,[S30] their efficacy is often dataset-dependent. In this instance, the traditional descriptor-based approach proved more

suitable for the chemical space of our dataset.

Applicability Domain

In any QSAR modeling, accurately defining the applicability domain (AD) is critical to understanding the reliability and confidence of the predictions generated by the model. In this study, the applicability domain was determined using Tanimoto similarity, calculated based on Morgan fingerprints. The similarity values were used to categorize the confidence level of each prediction into three distinct classes. Compounds with a Tanimoto similarity of 0.7 or greater to the training set were considered to fall within the 'high-confidence' domain, indicating robust predictive reliability. Compounds with a similarity score between 0.5 and 0.7 were classified as 'medium-confidence,' suggesting moderate reliability. Lastly, compounds with a similarity score below 0.5 were categorized as 'low-confidence,' where predictions may have reduced accuracy and should be interpreted with caution. This stratification of confidence levels provides an essential layer of transparency in evaluating the model's output and ensures that predictions are assessed based on their proximity to known training data, thereby aligning with best practices in QSAR model evaluation.

Team #8

Alexis Dougha

BFA, Université Paris Cité, CNRS UMR 8251, INSERM U1133, 75013 Paris, France

Data preprocessing

Salt-stripped SMILES were obtained from the original SMILES provided by the challenge organizers with rdkit.Chem.SaltRemover module from the RDKit library.[S4] No more pre-processing was performed.

Input features

198 molecular descriptors that are supposed to capture various important chemical, physical and topological properties of molecules were computed with RDKit. Descriptor calculation failed for 13 compounds, so 999 compounds out of the 1,012 initial compounds from the training dataset were used for model selection. Alternatively, we also generated 2,304 latent features with a graph transformer[S31] pre-trained on two million molecules from the ChEMBL29 dataset. Both the RDKit features and the latent features were combined with the benchmarked ML models to identify the best combinations of features and algorithms. Given the large number of latent features generated by the graph transformer, we hypothesized that feature selection could be beneficial. Therefore, we also combined the algorithms with a subset of 690 latent features selected with recursive feature elimination (based on random forest estimator to assess feature importance). Features were normalized with a MinMax scaler.

Splitting strategies

Two splitting strategies were used to perform hyperparameter optimization with grid search (train/test split). We expect that the models will perform differently when they are trained and tested on different data partitions.

- **Hard split:** This splitting strategy creates a test set dissimilar from the training set, making it more challenging for the algorithm to perform well. This splitting strategy is supposed to help to identify models that can reliably predict the endpoint of compounds dissimilar from the training set. The Morgan fingerprint of the compounds of the 999-compounds dataset were computed with RDKit (2,048 bits, radius of 2)[S32] and used to compute the Tanimoto similarity of every possible pair. Compounds with similarity to any other compound less than 1/3 act as the test set (225) and the other act as the training set (774).

- **Easy split:** This splitting strategy simply involves a random partition of the data, with the same number of compounds in each subset as for the hard splitting strategy (774 in training set, 225 in test set).

Model selection

With the aim of building a consensus model, we identified several promising models (among the ones with optimised hyperparameters) as the ones obtaining the smallest RMSE on the leaderboard set, which acts as a holdout test set to prevent overfitting. We decided to retain diverse algorithms, selected with hyperparameters optimized with the hard or the easy split, and with different types of input features. By doing so, we hope that each model will capture different patterns in the data, leading to a more robust consensus model.

Blind test prediction

The best models were retrained with the additional leaderboard data before predicting the activity of the blind set compounds.

Best performing models

Two Support Vector Machine (SVM)[S33] and one RF[S15] based models were chosen based on their RMSE on the leaderboard set and their diversity:

- SVM with rbf kernel, C=100, epsilon=0.5, latent features without feature selection
- RF with n_estimators=500, max_features=1/6, min_samples_split=6, RDKit features
- SVM with rbf, C=65, epsilon=1.5, latent features with feature selection

The hyperparameters of models a and c were optimized for the easy split while the hyperparameters of model b was optimized for the hard split. Since RF predictions are sensitive to random seed, an ensemble of 1,000 models was used.

Team #9

Luis Pinto

Independent Researcher. Montreal, Canada

In this competition, I developed a solution that combined an ensemble of four models: two MolFormer models,[S34] SMI-TED,[S35] and UniMol.[S36] This approach secured 9th place, achieving an RMSE of 21.4%. Notably, with a slightly different ensemble, my blind test set RMSE could have dropped to 20.4%, lower than the first place, but this lower submission was not selected as my final submission.

Data Preprocessing:

The molecules were cleaned using the molvs package,[S37] which led to some molecules sharing the same SMILES representation. To handle this, I averaged the target values for these duplicates, reducing the dataset to 1165 unique molecules. It might be beneficial to retain salt/neutralized pairs as separate data points, as their target values can vary significantly.

Model Training and Internal Test Set Creation:

After the release of leaderboard data, I retrained the models to incorporate this new information. I separated 20 molecules as an internal test set, with a final train set of 1145 molecules. Consistent 5-fold CV with the same seed and data ordering was applied across all models. The ensemble was built on the out-of-fold (OOF) predictions and validated on my internal test set.

Use of Auxiliary Targets:

To enhance the performance of the deep learning models, I incorporated the OOF predictions from tree-based models (LGBM[S23] and CatBoost[S7]) trained on different chemical descriptors as auxiliary targets. This approach allowed the deep learning models to learn from the implicit chemical knowledge captured by the tree-based models. However, this method is

prone to overfitting, making it essential to establish a robust CV strategy. Additionally, a strong correlation between CV scores and leaderboard performance is crucial to ensure that the models generalize well. Ideally, the leaderboard and test set distributions should align closely, which fortunately seemed to be the case in this competition. Training on these auxiliary targets did decrease the leaderboard RMSE (and OOF score) by a few points on each model individually.

Model-Specific Details:

MolFormer: Two versions of the MolFormer model were included in the ensemble. Both were pretrained on a subset of the ZINC dataset (approximately 50,000 data points closest to the Tox24 molecules using ECFP[S32]), Tox21, and Tox24 datasets using multitask regression (MTR) on 27 Mold2[S8] descriptors. This pretraining improved both CV and leaderboard performance by a few RMSE points.

- The first model was trained with the predictions of an LGBM model using OEState descriptors as auxiliary targets.
- The second model was trained with the predictions from a CatBoost model using Mold2 descriptors.

SMI-TED: This transformer model was fine-tuned without additional pretraining. It was trained with LGBM predictions using OEState[S24] descriptors as auxiliary target.

UniMol: The UniMol model was only fine-tuned and included in the final ensemble. It was trained with LGBM predictions using Mold2 descriptors as auxiliary targets.

Performance improvement:

The model above achieved an RMSE of 21.4, placing 9th on the leaderboard. However, for the consensus model presented in the main paper, a slight modification (removing the MolFormer model trained with CatBoost predictions) led to an improved RMSE of 20.4. This version was submitted during the competition, but was not the last one.

Team #10

Haobo Wang, Wenjia Liu

Key Laboratory of Industrial Ecology and Environmental Engineering (Ministry of Education), Dalian Key Laboratory on Chemicals Risk Control and Pollution Prevention Technology, School of Environmental Science and Technology, Dalian University of Technology, Dalian 116024, China

Overall Modeling Framework

This study constructed a transfer learning (TL) framework based on graph attention network (GAT) architecture[S38] for predicting chemical activity against transthyretin (TTR), designating datasets of chemical activity against thyroid hormone receptor β (TRβ) and TTR as source and target domains respectively. In addition, an ensemble strategy was employed to construct the TL-GAT models, enhancing the model robustness.

Data Preparation

The chemical TRβ activity dataset was collected from the PubChem database as the source domain dataset for the TL-GAT models, containing 2403 chemicals. The target domain dataset of chemical activity against TTR consists of training and leaderboard datasets reported by the Tox24 project, containing 1212 chemicals. In this study, the source and target domain datasets were randomly assigned as training, validation, and test sets in a ratio of 8:1:1 respectively.

Graph Attention Networks

GAT models take molecular graphs as input, where the feature vectors of the edges encode the features corresponding to chemical bonds, and the feature vectors of the nodes encode the features corresponding to non-hydrogen atoms. Hydrogen atoms are usually represented

as the number of hydrogen atoms attached to the atom in the non-hydrogen atom feature vectors.

During the training, an attention weight parameter (P_{AW}) allows the GAT model to focus on the important information of the training samples and learn them fully. The attention mechanism adds P_{AW} to the feature aggregation process so that the model can identify the important nodes in the molecular graphs and gradually increase the P_{AW} of the important nodes by back propagation and gradient descent algorithm. At the same time, the attention mechanism also makes the P_{AW} of the nodes that contribute less to the prediction gradually decrease to avoid their negative interference.

Model Construction

The GAT backbone leverages attention mechanisms to capture relationships between end-points to be predicted and inputs of molecular graphs to achieve "end-to-end" learning. In the first stage, the GAT models were pre-trained on the source domain dataset. In the second stage, the pre-trained GAT model with the highest determination coefficient (R^2) on the test set was employed to initialize learnable parameters of GAT models in the fine-tuning process.

During the fine-tuning, the hyperparameters were optimized to control the extent of knowledge transfer and achieve improved model performance. The target domain dataset was randomly divided into training, validation, and test sets 20 times, and the TL-GAT models with R^2 on the training sets over 0.9, considered well-established, were stored as an ensemble. The predicted values from the models within the ensemble were averaged to improve robustness of the models.

AD characterization

An AD characterization method abbreviated as $\mathrm{AD_{SAL}}\{\rho_{s,q} \geq \rho_{s,T}, I_{A,q} \leq I_{A,T}\}$ was launched in the current study, with similarity density (ρ_{s}) to characterize chemical space of training

data, and inconsistency of activity (I_A) to warn the emergence of activity cliffs (ACs).[S39] A query compound with a lower ρ_s value implies it has fewer structurally similar training chemicals than compounds with higher ρ_s values. $\rho_\mathrm{s,T}$ and $I_{A,T}$ are the corresponding thresholds. A query compound with a large I_A value implies the compound is likely to be located at the ACs. Therefore, molecules with small ρ_s and/or large I_A values tend to lie outside the ADs. ρ_s defines weighted similarity density between a query compound and training compounds:

$$\rho_{s,q} = \sum_{t \in T} w_{q,t} \tag{1}$$

where the subscript q and t represent a query compound and a training compound; T represents the entire training set; and $w_{q,t}$ stands for weight function for the pair of compound q and t, calculated as

$$w_{q,t} = \exp\left(-a \times \frac{1 - S_{Mq,t}}{S_{Mq,t} + \epsilon}\right) \tag{2}$$

where $S_{Mq,t}$ stands for pairwise molecular similarity between compound q and t; a is a parameter that modulates weighted contribution of training compounds, which was set to 10; is an infinitesimal quantity to ensure that the denominator is not zero, which was set to 1×10^{-6}. In the current study, I_A was calculated to quantify weighted inconsistency of activity between a query compound (q) and training compounds (t $\in$ T):

$$I_{A,q} = \frac{\sum_{t \in T} w_{q,t} \cdot S_{WD,t}}{\sum_{t \in T} w_{q,t}} \tag{3}$$

where $S_{WD,t}$ stands for weighted local discontinuity scores (S_{WD}) for the training compound (t). $S_{WD,t}$ was proposed to be calculated as

$$S_{WD,t} = \frac{\sum_{v|v \in T, t \neq v} w_{t,v} \cdot S_{M,t,v} \cdot |y_t - y_v|}{\sum_{v|v \in T, t \neq v} w_{t,v}} \tag{4}$$

where the subscript t and v represent two training compounds; the weight function for

the pairwise compound t and v ($w_{t,v}$ was calculated with either eq. (2); y_t and y_v stand for observed labels.

Team #11

Vladimir Chupakhin

Cheminformatics Solutions, Simulations Plus. Lancaster, CA 93534 United States

Data

Since transthyretin (TTR) is an understudied target, we developed a feature set by leveraging models for related targets. We identified them from the Protein Data Bank that bind to compounds structurally similar to thyroxine and retinol, and cross-referenced these targets with PubChem[S40] and ChEMBL[S12] e.g. plasma retinol-binding protein. TTR protein structures revealed that the TTR binding site is β-sheet enriched, thus we included amyloid, tau and prions as related targets. Additionally, a literature review uncovered a set of thyroid-related toxicity studies for iodothyronine deiodinases, thyrotropin-releasing hormone receptor, thyroid-stimulating hormone receptor, thyroperoxidase and sodium/iodide symporter, whose associated data were incorporated into the auxiliary data pool. This led to the creation of AUX, a set of 180 features derived from classification (e.g. active/inactive) or regression models (Ki, IC50, %) based on various descriptors (up to 4). Before modeling all compounds went through the established RDKit-based pipeline for compound standardization.[S4]

Modeling

Models composing AUX descriptors were based on RDKit 2D,[S4] Mordred 2D[S20] and RDKit Morgan fingerprint (R=3, 2048 bits).[S32] 5-fold CV was used as a default modeling setup for most of the developed models. For the initial set of models for TTR we used the CatBoost Regressor algorithm[S7] with the following descriptors: AUX, RDKit 2D, Mordred 2D, ChemBERTA,[S41] Reduced Graph Fingerprint,[S42] and RDKit Morgan fingerprints with varying radii and folded sizes. We selected the top four descriptors - AUX, Mordred 2D, RDKit 2D, and Morgan fingerprint-based on their RMSE rankings for ensemble modeling. Utilizing the

scikit-learn framework, we exhaustively tested combinations of these descriptors with either the Voting or Stacking Regressor.[S43] The most effective heterogeneous ensemble consisted of CatBoost Regressor models based on Mordred 2D and AUX descriptors combined with a VotingRegressor as a final model. To further enhance performance, we introduced an ensemble involving 50 distinct VotingRegressor models with multiple random initializations with the median of those predictions as our final submission. Insights gained from this approach will be considered for future integration into the ADMET Predictor platform by Simulations Plus, Inc. A full list of used sources and references will be provided upon request.

References

(S1) Tetko, I. V. Tox24 Challenge. *Chemical Research in Toxicology* **2024**, *37*, 825–826.

(S2) Makarov, D. M.; Ksenofontov, A. A.; Budkov, Y. A. Consensus Modeling for Predicting Chemical Binding to Transthyretin as the Winning Solution of the Tox24 Challenge. *Chemical Research in Toxicology* **2025**, *38*, 392–399.

(S3) Sushko, I.; Novotarskyi, S.; Körner, R.; Pandey, A. K.; Rupp, M.; Teetz, W.; Brandmaier, S.; Abdelaziz, A.; Prokopenko, V. V.; Tanchuk, V. Y.; Todeschini, R.; Varnek, A.; Marcou, G.; Ertl, P.; Potemkin, V.; Grishina, M.; Gasteiger, J.; Schwab, C.; Baskin, I. I.; Palyulin, V. A.; Radchenko, E. V.; Welsh, W. J.; Kholodovych, V.; Chekmarev, D.; Cherkasov, A.; Aires-de Sousa, J.; Zhang, Q.-Y.; Bender, A.; Nigsch, F.; Patiny, L.; Williams, A.; Tkachenko, V.; Tetko, I. V. Online chemical modeling environment (OCHEM): web platform for data storage, model development and publishing of chemical information. *Journal of Computer-Aided Molecular Design* **2011**, *25*, 533–554.

(S4) Landrum, G. RDKit: Open-source cheminformatics. 2013; `http://www.rdkit.org`.

(S5) De Oliveira, C.; Yu, H. S.; Chen, W.; Abel, R.; Wang, L. Rigorous Free Energy

Perturbation Approach to Estimating Relative Binding Affinities between Ligands with Multiple Protonation and Tautomeric States. *Journal of Chemical Theory and Computation* **2019**, *15*, 424–435.

(S6) Tetko, I. V.; M. Lowe, D.; Williams, A. J. The development of models to predict melting and pyrolysis point data associated with several hundred thousand compounds mined from PATENTS. *Journal of Cheminformatics* **2016**, *8*, 2.

(S7) Prokhorenkova, L.; Gusev, G.; Vorobev, A.; Dorogush, A. V.; Gulin, A. CatBoost: unbiased boosting with categorical features. *Advances in neural information processing systems* **2018**, *31*.

(S8) Hong, H.; Xie, Q.; Ge, W.; Qian, F.; Fang, H.; Shi, L.; Su, Z.; Perkins, R.; Tong, W. Mold ^{2} , Molecular Descriptors from 2D Structures for Chemoinformatics and Toxicoinformatics. *Journal of Chemical Information and Modeling* **2008**, *48*, 1337–1344.

(S9) Reiser, P.; Eberhard, A.; Friederich, P. Graph neural networks in TensorFlow-Keras with RaggedTensor representation (kgcnn). *Software Impacts* **2021**, *9*, 100095.

(S10) Yang, K.; Swanson, K.; Jin, W.; Coley, C.; Eiden, P.; Gao, H.; Guzman-Perez, A.; Hopper, T.; Kelley, B.; Mathea, M.; others Analyzing learned molecular representations for property prediction. *Journal of chemical information and modeling* **2019**, *59*, 3370–3388.

(S11) Karpov, P.; Godin, G.; Tetko, I. V. Transformer-CNN: Swiss knife for QSAR modeling and interpretation. *Journal of Cheminformatics* **2020**, *12*, 17.

(S12) Gaulton, A.; Bellis, L. J.; Bento, A. P.; Chambers, J.; Davies, M.; Hersey, A.; Light, Y.; McGlinchey, S.; Michalovich, D.; Al-Lazikani, B.; Overington, J. P. ChEMBL: a large-scale bioactivity database for drug discovery. *Nucleic Acids Research* **2012**, *40*, D1100–D1107.

(S13) Makarov, D. M.; Fadeeva, Y. A.; Shmukler, L. E.; Tetko, I. V. Machine learning models for phase transition and decomposition temperature of ionic liquids. *Journal of Molecular Liquids* **2022**, *366*, 120247.

(S14) Tetko, I. V.; Sushko, I.; Pandey, A. K.; Zhu, H.; Tropsha, A.; Papa, E.; Oberg, T.; Todeschini, R.; Fourches, D.; Varnek, A. Critical assessment of QSAR models of environmental toxicity against Tetrahymena pyriformis: focusing on applicability domain and overfitting by variable selection. *Journal of Chemical Information and Modeling* **2008**, *48*, 1733–1746.

(S15) Breiman, L. Random Forests. *Machine Learning* **2001**, *45*, 5–32.

(S16) Chawla, N. V.; Bowyer, K. W.; Hall, L. O.; Kegelmeyer, W. P. SMOTE: Synthetic Minority Over-sampling Technique. *Journal of Artificial Intelligence Research* **2002**, *16*, 321–357.

(S17) Mirjalili, S.; Mirjalili, S. Genetic algorithm. *Evolutionary algorithms and neural networks: theory and applications* **2019**, 43–55.

(S18) Durant, J. L.; Leland, B. A.; Henry, D. R.; Nourse, J. G. Reoptimization of MDL keys for use in drug discovery. *Journal of chemical information and computer sciences* **2002**, *42*, 1273–1280.

(S19) He, K.; Zhang, X.; Ren, S.; Sun, J. Deep residual learning for image recognition. Proceedings of the IEEE conference on computer vision and pattern recognition. 2016; pp 770–778.

(S20) Moriwaki, H.; Tian, Y.-S.; Kawashita, N.; Takagi, T. Mordred: a molecular descriptor calculator. *Journal of Cheminformatics* **2018**, *10*, 4.

(S21) Kurosaki, K.; Wu, R.; Uesawa, Y. A Toxicity Prediction Tool for Potential Ago-

nist/Antagonist Activities in Molecular Initiating Events Based on Chemical Structures. *International Journal of Molecular Sciences* **2020**, *21*, 7853.

(S22) Chen, T.; Guestrin, C. Xgboost: A scalable tree boosting system. Proceedings of the 22nd acm sigkdd international conference on knowledge discovery and data mining. 2016; pp 785–794.

(S23) Ke, G.; Meng, Q.; Finley, T.; Wang, T.; Chen, W.; Ma, W.; Ye, Q.; Liu, T.-Y. LightGBM: a highly efficient gradient boosting decision tree. Proceedings of the 31st International Conference on Neural Information Processing Systems. Red Hook, NY, USA, 2017; pp 3149–3157, event-place: Long Beach, California, USA.

(S24) Hall, L. H.; Kier, L. B. Electrotopological state indices for atom types: a novel combination of electronic, topological, and valence state information. *Journal of Chemical Information and Computer Sciences* **1995**, *35*, 1039–1045.

(S25) Huuskonen, J. J.; Livingstone, D. J.; Tetko, I. V. Neural network modeling for estimation of partition coefficient based on atom-type electrotopological state indices. *Journal of chemical information and computer sciences* **2000**, *40*, 947–955.

(S26) Willighagen, E. L.; Mayfield, J. W.; Alvarsson, J.; Berg, A.; Carlsson, L.; Jeliazkova, N.; Kuhn, S.; Pluskal, T.; Rojas-Chertó, M.; Spjuth, O.; others The Chemistry Development Kit (CDK) v2. 0: atom typing, depiction, molecular formulas, and substructure searching. *Journal of cheminformatics* **2017**, *9*, 1–19.

(S27) Eytcheson, S. A.; Tetko, I. V. Which modern AI methods provide accurate predictions of toxicological endpoints? Analysis of Tox24 challenge results. **2025**,

(S28) Hemmerich, J. {KNIME} Structure Standardisation Workflow. 2020; `github.com/PharminfoVienna/Chemical-Structure-Standardisation`.

(S29) Zakharov, A. V.; Zhao, T.; Nguyen, D.-T.; Peryea, T.; Sheils, T.; Yasgar, A.; Huang, R.; Southall, N.; Simeonov, A. Novel Consensus Architecture To Improve Performance of Large-Scale Multitask Deep Learning QSAR Models. *Journal of Chemical Information and Modeling* **2019**, *59*, 4613–4624.

(S30) Jain, S.; Siramshetty, V. B.; Alves, V. M.; Muratov, E. N.; Kleinstreuer, N.; Tropsha, A.; Nicklaus, M. C.; Simeonov, A.; Zakharov, A. V. Large-Scale Modeling of Multispecies Acute Toxicity End Points Using Consensus of Multitask Deep Learning Methods. *Journal of Chemical Information and Modeling* **2021**, *61*, 653–663.

(S31) Li, H.; Zhang, R.; Min, Y.; Ma, D.; Zhao, D.; Zeng, J. A knowledge-guided pre-training framework for improving molecular representation learning. *Nature Communications* **2023**, *14*, 7568.

(S32) Rogers, D.; Hahn, M. Extended-Connectivity Fingerprints. *Journal of Chemical Information and Modeling* **2010**, *50*, 742–754.

(S33) Suthaharan, S.; Suthaharan, S. Support vector machine. *Machine learning models and algorithms for big data classification: thinking with examples for effective learning* **2016**, 207–235.

(S34) Ross, J.; Belgodere, B.; Chenthamarakshan, V.; Padhi, I.; Mroueh, Y.; Das, P. Large-scale chemical language representations capture molecular structure and properties. *Nature Machine Intelligence* **2022**, *4*, 1256–1264.

(S35) Soares, E.; Shirasuna, V.; Brazil, E. V.; Cerqueira, R.; Zubarev, D.; Schmidt, K. A Large Encoder-Decoder Family of Foundation Models For Chemical Language. 2024; `https://arxiv.org/abs/2407.20267`, Version Number: 1.

(S36) Zhou, G.; Gao, Z.; Ding, Q.; Zheng, H.; Xu, H.; Wei, Z.; Zhang, L.; Ke, G. Uni-Mol: A Universal 3D Molecular Representation Learning Framework. 2022; `https://chemrxiv.org/engage/chemrxiv/article-details/628e5b4d5d948517f5ce6d72`.

(S37) Swain, M. MolVS: molecule validation and standardization. *Web Page* **2018**,

(S38) Wang, H.; Wang, Z.; Chen, J.; Liu, W. Graph Attention Network Model with Defined Applicability Domains for Screening PBT Chemicals. *Environmental Science & Technology* **2022**, *56*, 6774–6785.

(S39) Wang, H.; Liu, W.; Chen, J.; Wang, Z. Applicability Domains Based on Molecular Graph Contrastive Learning Enable Graph Attention Network Models to Accurately Predict 15 Environmental End Points. *Environmental Science & Technology* **2023**, *57*, 16906–16917.

(S40) Kim, S.; Chen, J.; Cheng, T.; Gindulyte, A.; He, J.; He, S.; Li, Q.; Shoemaker, B. A.; Thiessen, P. A.; Yu, B.; Zaslavsky, L.; Zhang, J.; Bolton, E. E. PubChem in 2021: new data content and improved web interfaces. *Nucleic Acids Research* **2021**, *49*, D1388–D1395.

(S41) Chithrananda, S.; Grand, G.; Ramsundar, B. ChemBERTa: Large-Scale Self-Supervised Pretraining for Molecular Property Prediction. 2020; `https://arxiv.org/abs/2010.09885`, Version Number: 2.

(S42) Stiefl, N.; Watson, I. A.; Baumann, K.; Zaliani, A. ErG: 2D Pharmacophore Descriptions for Scaffold Hopping. *Journal of Chemical Information and Modeling* **2006**, *46*, 208–220.

(S43) Wolpert, D. H. Stacked generalization. *Neural Networks* **1992**, *5*, 241–259.
