# Supplementary material for: Consensus Modeling Strategies for Predicting Transthyretin Binding Affinity from Tox24 Challenge Data
Source: Chem Res Toxicol. 2025 May 15;38(6):1061–71. doi: 10.1021/acs.chemrestox.5c00018 (PMC12175157; doi:10.1021/acs.chemrestox.5c00018)
Supplement: Supplementary file 3 [file tx5c00018_si_003.pdf]

Supplementary Information 3

Consensus Modeling Strategies for Predicting Transthyretin Binding Affinity from Tox24 Challenge data

Thalita Cirino,[*,†] Luis Pinto,[‡] Mateusz Iwan,[¶] Alexis Dougha,[§] Bono Lučić,[‖] Antonija Kraljević,[⊥] Zaven Navoyan,[#] Ani Tevosyan,[#] Hrach Yeghiazaryan,[#] Lusine Khondkaryan,[#,@] Narek Abelyan,[△] Vahe Atoyan,[#] Nelly Babayan,[#,@] Yuma Iwashita,[∇] Kyosuke Kimura,[∇] Tomoya Komasaka,[∇] Koki Shishido,[∇] Taichi Nakamura,[∇] Mizuho Asada,[∇] Sankalp Jain,[††] Alexey V. Zakharov,[††] Haobo Wang,[‡‡] Wenjia Liu,[‡‡] Vladimir Chupakhin,[¶¶] and Yoshihiro Uesawa[∇]

†*Molecular Biotechnology and Health Sciences Department, University of Turin, 10126 Turin, Italy*

‡*Independent Researcher. Montreal, Canada*

¶*Mario Negri Institute for Pharmacological Research IRCCS, 20156 Milan, Italy*

§*BFA, Université Paris Cité, CNRS UMR 8251, Inserm U1133, 75013 Paris, France*

‖*Ruđer Bosković Institute, 10000 Zagreb, Croatia*

⊥*Faculty of Mechanical Engineering, Computing and Electrical Engineering, University of Mostar, 88000 Mostar, Bosnia and Herzegovina*

#*Toxometris.ai. Glendale, CA 91204 United States*

@*Institute of Molecular Biology, NAS RA. Yerevan 0014, Armenia*

△*Biocentric.ai. Yerevan 0075, Armenia*

∇*Department of Medical Molecular Informatics, Meiji Pharmaceutical University. Tokyo 204-8588, Japan*

††*National Center for Advancing Translational Sciences (NCATS–NIH). Rockville, MD 20850 USA*

‡‡*School of Environmental Science and Technology, Dalian University of Technology. Dalian 116024, China*

¶¶*Cheminformatics Solutions, Simulations Plus. Lancaster, CA 93534 USA*

E-mail: thalita.cirino@unito.it

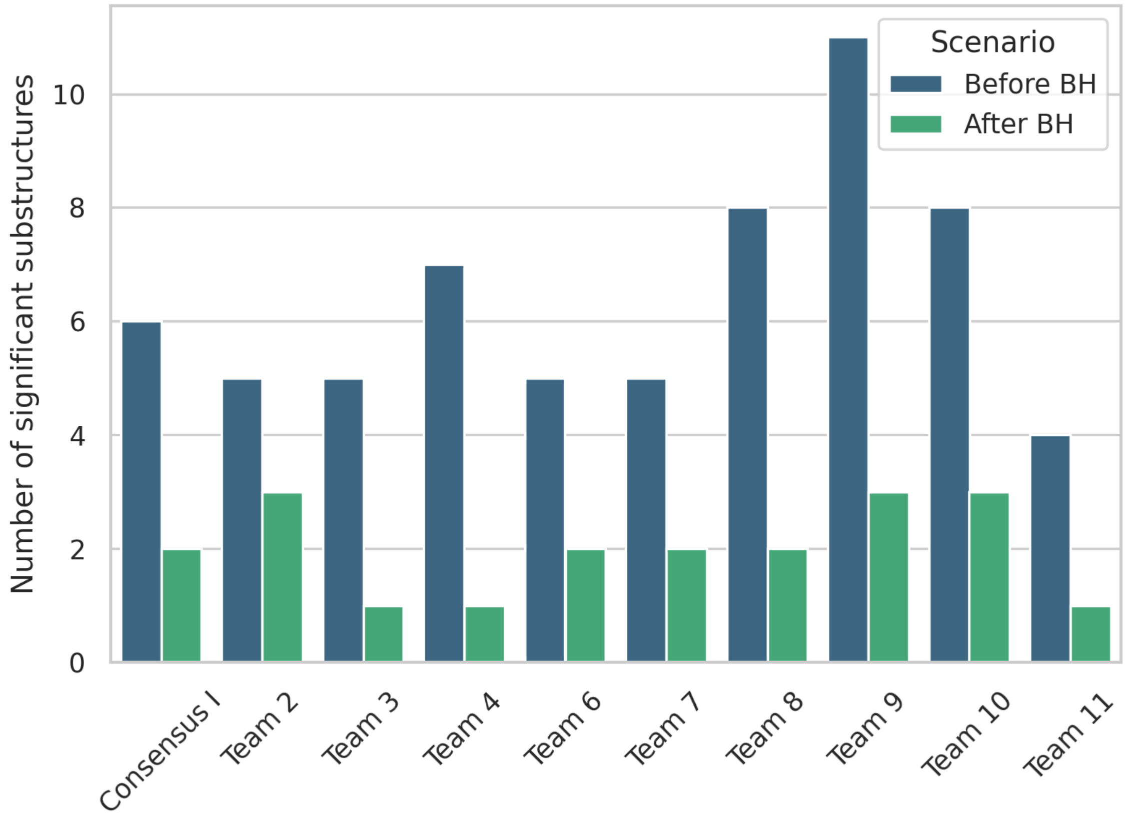

Figure S3-A: Numbers of significant substructures before and after applying the Benjamini-Hochberg procedure for each model.

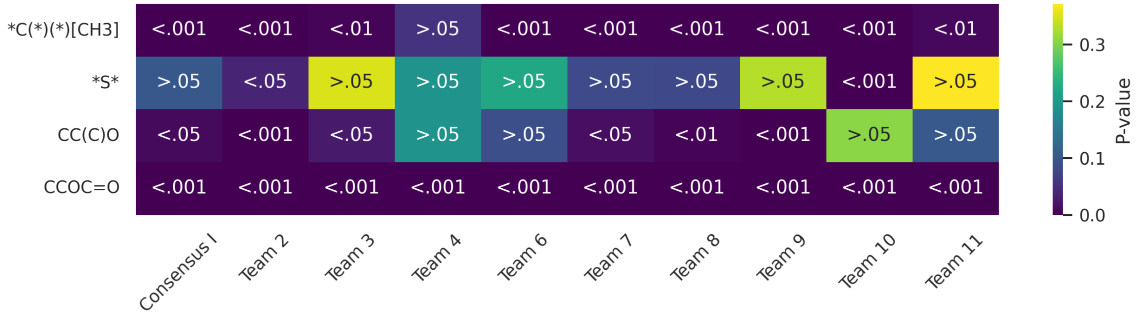

Figure S3-B: Fragments significantly affecting the quality of predictions and calculated p-values.

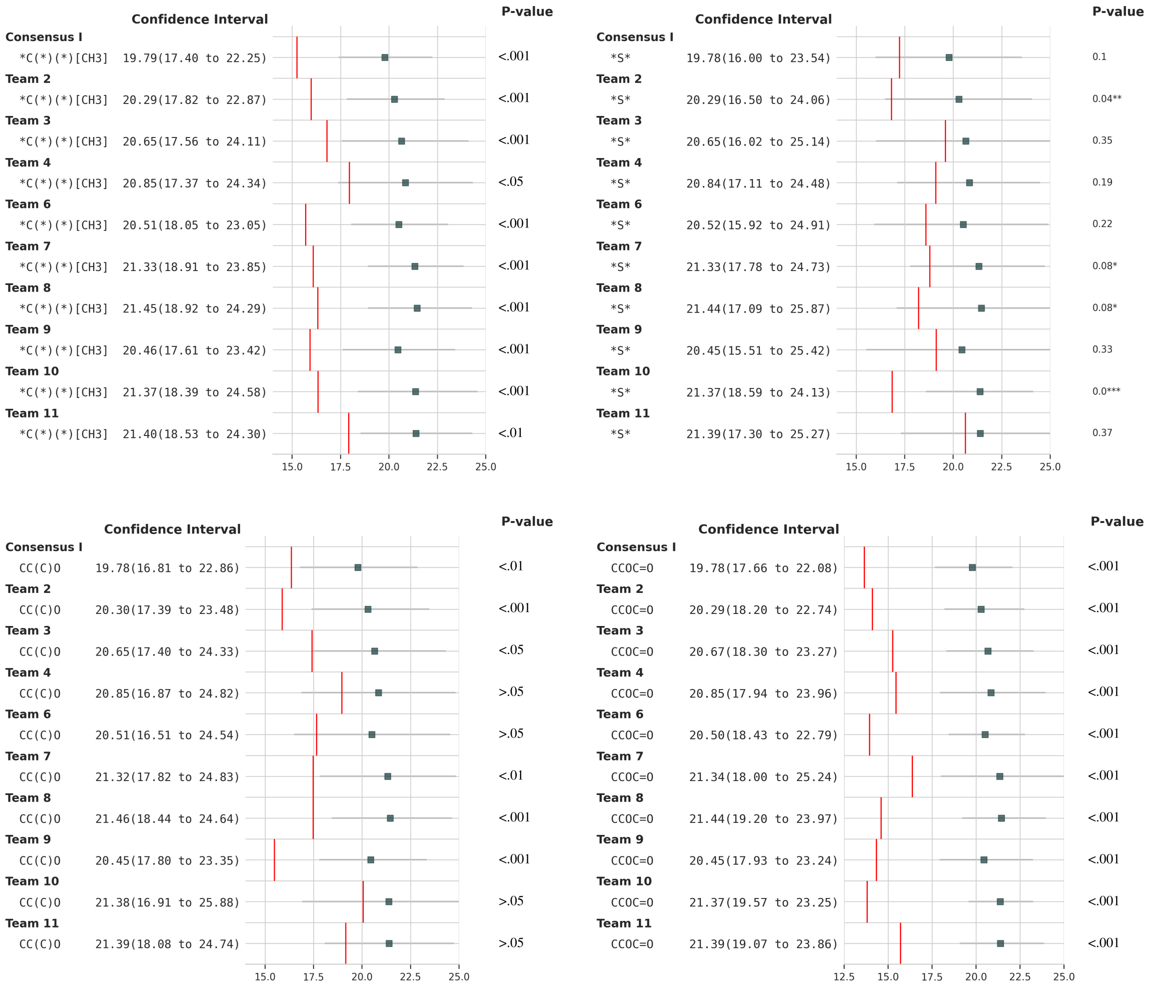

Figure S3-C: 95% Confidence Intervals for each statistically significant fragment. RMSE for each subset of the test dataset is shown as a red line. * p-value < 0.1, ** p-value < 0.05, *** p-value < 0.01.
